# Integration of genomics, clinical characteristics and baseline biological profiles to predict the risk of liver injury induced by high-dose methotrexate

**DOI:** 10.3389/fphar.2024.1423214

**Published:** 2024-11-28

**Authors:** Chenquan Lin, Rui Ma, Xiao Zeng, Bikui Zhang, Ting Cao, Shimeng Jiao, Hui Chen, Yifang He, Mouze Liu, Hualin Cai

**Affiliations:** ^1^ Department of Pharmacy, The Second Xiangya Hospital of Central South University, Changsha, China; ^2^ Institute of Clinical Pharmacy, Central South University, Changsha, China; ^3^ International Research Center for Precision Medicine, Transformative Technology and Software Services, Changsha, Hunan, China

**Keywords:** methotrexate, drug induced liver injury, therapeutic drug monitoring, pharmacogenomics, nomogram

## Abstract

**Background:**

High-dose methotrexate (HD-MTX) is commonly employed in the treatment of malignant tumors in children and young adults due to its distinctive therapeutic efficacy. Nonetheless, the systemic exposure to MTX often results in liver injury (drug induced liver injury, DILI), thereby imposing limitations on the sustained administration of HD-MTX. Additionally, individual variations including genetic underpinnings attributable to disparities in therapeutic effects and clinical toxicity remain to be elucidated.

**Methods:**

A total of 374 patients receiving initial HD-MTX treatment were selected for this study, which aimed to establish a predictive model using binary logistic regression and a visual nomogram for DILI risk assessment. Demographic and clinical characteristics were collected at baseline and post-HD-MTX to explore their correlations with the occurrence of DILI. Additionally, genotyping of 25 single nucleotide polymorphisms from drug transporters and enzymes in the folic acid cycle was performed.

**Result:**

G allele mutation in *ABCB1* rs1128503, *1b/*1b and *1b/*15 haplotypic mutation in *SLCO1B1*, female gender, and MTX dosage were identified as independent factors for moderate/severe DILI. Patients with GA or AA genotype in ABCB1 rs1128503 showed significant higher 24h MTX concentration than GG, and those with *1b/*1b haplotype group in SLCO1B1 exhibited lower dose adjusted concentration (C/D) than *1a/*1a group. Besides, patient administrated with HD-MTX were more prevalent to have higher C/D levels when using intravenous plus triple intrathecal injection route than those who were using intravenous injection alone. The composite predictive model (ROC curve: AUC = 0.805), comprising above four factors and 24h MTX concentration, exhibited high accuracy.

**Conclusion:**

Female gender, recessive mutation in ABCB1 rs1128503, and a range of MTX concentration may be risk factors for increased susceptibility to DILI. Conversely, the *1b/*1b and *1b/*15 mutations in *SLCO1B1* may have a protective effect against DILI. The proposed predictive model facilitates early individual risk assessment, enabling the implementation of proactive prevention strategies.

## 1 Introduction

Malignant tumors have emerged as one of the leading causes of mortality in childhood, adolescence, and young adulthood. The predominant cancer types during these periods include acute lymphoblastic leukemia (ALL), tumors of the central nervous system (CNS), and non-Hodgkin lymphomas (NHL) (Steliarova-Foucher et al., 2017). Additionally, osteosarcoma (OS), the primary bone tumor, predominantly affects adolescents and young adults, constituting approximately 5% of all childhood cancers (Hameed and Mandelker, 2018). High dose methotrexate (HD-MTX), with dosage of MTX higher than 20 mg/kg or 500 mg/m^2^, has demonstrated efficacy in the treatment of a variety of childhood cancers, including ALL, NHL, OS, and specific high-grade tumors of CNS (Daetwyler et al., 2022). Although the high dose of MTX serves as a therapeutic dose, it also presents a potentially lethal risk including myelosuppression, mucositis, and drug-induced liver injury (DILI), thereby constraining its clinical application for solid tumors.

The extracellular-to-intracellular journey of MTX involves various transporters and active enzymes. Initially, MTX enters the cell through an active transporter mediated by the reduced folate carrier (*RFC1/SLC19A1*) (Wang et al., 2014). Subsequently, the intracellular MTX content undergoes dynamic regulation through the actions of *FPGS* and *GGH* (Fotoohi and Albertioni, 2008). Firstly, *FPGS* polyglutamates MTX into MTX-PG, the active form that directly inhibits enzymes in the folate cycle, such as *DHFR* and thymidylate synthase (*TYMS*) (Faganel Kotnik et al., 2011). Moreover, the folate cycle related to the action metabolism of MTX is intricate, involving several additional enzymes, including *MTHFR*, methylenetetrahydrofolate dehydrogenase (*MTHFD1*) and 5-aminoimidazole-4-carboxamide ribonucleotide formyl transferase (gene symbol *ATIC*) (Samara et al., 2014). Secondly, polyglutamylation of MTX can be reversed by hydrolysis via *GGH*, facilitating the cellular efflux of MTX and short-chained MTX-PG (Swerts et al., 2006), which can be released into the bloodstream. Finally, various transporters, including those of the ABC family (e.g., multidrug resistance protein *ABCB1* and multi-drug resistance-related protein *ABCC2*) (Treviño et al., 2009), and organic anion transporters such as *SLCO1B1* (Hoed et al., 2015), pump MTX out of the cells. Moreover, apart from genetic factors, patients with malignant tumors often require non-steroidal anti-inflammatory drugs (NSAIDs) for managing disease flares and chronic pain, proton pump inhibitors (PPIs) for gastroprotection, or antibiotics like sulfamethoxazole (SMZ) to address infections (Hall et al., 2017). The competition between these agents and therapeutic drugs for transporters, along with potential synergistic toxicities, can significantly impact the pharmacokinetics of MTX and exacerbate drug-induced liver injury (DILI).

The potential mechanism under the DILI from HD-MTX is still not clear. But its cytotoxicity seems closely related to the inhibition to folic acid synthesis and the adenosine signaling (Brown et al., 2016). For one hand, the higher accumulation of MTX polyglutamates (MTX-PG), the metabolite of MTX in cells, can initiate intracellular pathological processes associated with inflammation, oxidative stress, fibrosis, and apoptosis (Ezhilarasan, 2021). Since only the non-polyglutamate form of MTX can return to the extracellular environment, the toxicity arising from elevated MTX-PG levels may manifest through alterations in the activity of transformational enzymes and in the function of transporters. For the other hand, MTX (specifically MTX-PG) interferes with the activity of AICAR transformylase (ATIC), leading to an increase in the concentration of AMP and adenosine both intracellular and extracellular (Chan and Cronstein, 2010). The release of adenosine stimulates the activation of stellate cells in the liver, which in turn promotes fibrogenesis (O’Dell, 2004). Due to the importance of enzymes and transporters in the liver toward the intracellular and extracellular levels of MTX and its metabolite, their genetic polymorphism may reflect individual differences in the clinical response and toxicity of patients during the MTX therapy (Joannon et al., 2004). Therefore, the objective of this study is to establish a diagnostic predictive model integrating the MTX related transporter gene polymorphisms with these clinical characteristics, aiming to preempt toxicity occurrences in the early stages of hospitalization.

## 2 Materials and methods

### 2.1 Ethics statement

This study was approved by the Ethics Committee of the Second Xiangya Hospital of Central South University, and was registered at the Chinese Clinical Trial Registry online (ChiCTR2200065150). We obtained the informed consents from patients or their guardians before inclusion, and collected the clinical data from medical records systems. All study procedures adhered to the guidelines of the Helsinki Declaration (World Medical Association, 2013).

### 2.2 Participants and data collection

A total of 374 patients receiving MTX treatment were enrolled from the Second Xiangya Hospital of Central South University from December 2019, to July 2022. The inclusion criteria were (1) hospitalized patients diagnosed with ALL, NHL, OS or Langerhans’cell histiocytosis; (2) patients who accepted their first treatment of HD-MTX in hospital or off HD-MTX at least 1 month before admission; (3) ages from 0 to 65; (4) therapeutic drug monitoring throughout the entire chemotherapy regimen with HD-MTX including at least one time monitoring point (24h, 48h, 72 h) after initial administration. The exclusion criteria were (1) pre-existing hepatitis, cirrhosis, or other liver diseases before treatment; (2) discontinuation of HD-MTX treatment due to severe adverse reactions or other reasons; (3) incomplete patient cases in the medical record system.

Retrospective data for model development were extracted from the medical record system before and after treatment, including demographic data (age, sex, BMI, hospitalization, diagnosis), physiological and biochemical parameters (serum creatinine (Scr), white blood cell count (WBC), blood platelet count (PLT), alanine aminotransferase (ALT) and aspartate transaminase (AST)), and pharmacokinetic data (methotrexate dosage, administration, duration, drugs interactions). Liver functional indicators were used to determine severity of DILI based on hepatic failure grading in Common Terminology Criteria for Adverse Events 5.0 (CTCAE 5.0) part “Investigation” (Yamamoto et al., 2021). The severity of DILI was determined based on ALT/AST levels: (1) Healthy: ALT or AST < upper limit of normal value (ULN) if baseline was normal; (2) mild: >ULN - 3.0 x ULN if baseline was normal; >1.5 - 3.0 x baseline if baseline was abnormal; (3) moderate: >3.0 - 5.0 x ULN if baseline was normal; >3.0 - 5.0 x baseline if baseline was abnormal; (4) severe: >5.0 - 20.0 x ULN if baseline was normal; >5.0 - 20.0 x baseline if baseline was abnormal; (5) extremely severe: >20.0 x ULN if baseline was normal; >20.0 x baseline if baseline was abnormal. The subsequent objective was to explore the potential correlations among MTX blood concentration, liver toxicity, and various genotypes.

### 2.3 Administration and concentration monitoring of methotrexate

In the treatment of hematologic malignancies like ALL and NHL, MTX is initially administered as a total dose ranging from 1 to 8 g/m^2^. Besides, the loading dose, accounting for one-tenth of the total, is delivered through intravenous infusion over 0.5 h. The subsequent maintenance dose, constituting the remaining nine-tenths of the total, is administered over an extended duration (Pauley et al., 2013). And for treatment of Langerhans cell histiocytosis, it consisted of an intravenous infusion of 1–2 g/m^2^ MTX for 24 h. In contrast, for the treatment of OS, MTX is administered intravenously at a dosage of 8–12 g/m^2^, with a rapid infusion period lasting 4–6 h (National Health Commission of the People’s Republic of China, 2019). In the 24 h infusion regimen, the first dose of leucovorin was administrated at 15 mg/m^2^ every 6 h at 36–44 h after the start of HDMTX infusion while in the rapid infusion regimen, it was administrated earlier at 12–24 h. Subsequently, the following dose of leucovorin should be adjusted based on the concentration of MTX at 42h–48 h (C_42–48h_: 0.2–1.0 µM, leucovorin: 15 mg/m^2^, q6h; C_42–48h_: 1.0–2.0 µM, leucovorin: 30 mg/m^2^, q6h; C_42–48h_: 2.0–3.0 µM, leucovorin: 45 mg/m^2^, q6h; C_42–48h_: 3.0–4.0 µM, leucovorin: 60 mg/m^2^, q6h; C_42–48h_: 4.0–5.0 µM, leucovorin: 75 mg/m^2^, q6h) and administration should continue until the concentration falls below 0.1–0.2 µM (Song et al., 2022). To prevent meningeal tumors, patients also received intrathecal injection chemotherapy of 10 mg methotrexate, 30 mg cytarabine and 5 mg dexamethasone once a week. Thereafter, the subsequent dose was adjusted by clinicians based on clinical reactions and results of therapeutic drug monitoring.

Blood samples were collected from patients receiving HD-MTX or intrathecal methotrexate at 24, 48, and 72 h after each course/cycle. Each venous blood sample (2 mL) was collected into anticoagulant tubes and stored in a −80°C refrigerator (DW-86L578J). All methotrexate plasma concentrations were analyzed by automatic two-dimensional liquid chromatography (2D-HPLC, Demeter Instrument Co., Ltd., Hunan, China). In this study, the safety range of methotrexate concentration was 24 h ≤ 10 μmol L^−1^ (4–6 h infusion), 16 μmol L^−1^ ≤ 24 h ≤ 1 μmol L^−1^ (24 h infusion), 48 h ≤ 1 μmol L^−1^ and 72 h ≤ 0.1 μmol L^−1^ (4–6 h infusion and 24 h infusion). When the post-treatment concentration of MTX is beyond the secure range in these three time points, it can be defined as the 24h, 48h, or 72 h clearance delay.

### 2.4 Genotyping

The blood samples obtained from biobank were used to extract the total DNAs, and single nucleotide polymorphisms (SNPs) of crucial transporter on cell membranes and enzymes in the folic acid cycle, which are relevant to the distribution and metabolism of methotrexate. The Pharmacogenetics and Pharmacogenomics Knowledge Base (https://www.pharmgkb.org/) and 1,000 genomes project (1KGP) (https://www.internationalgenome.org/) were employed to determine the genomic location, subcellular location and population frequency of targeted SNP polymorphisms. After a comprehensive consideration of allele frequency, sample size and clinical evidence level in East Asian population, we selected 25 SNPs as our target gene sequence (Table 2).

The process began with the extraction of deoxyribonucleic acid (DNA) from whole blood samples (1–3 mL) using the commercially available EZNA^®^ SQ. Blood DNA Kit II. Subsequently, the concentration of DNA samples was measured using the Qubit^®^ 3.0 Fluorometer (Thermo, Q33216). The process of SNP detection involved the integration of multiple PCR techniques, single-nucleotide extension technology, and Matrix-Assisted Laser Desorption/Ionization–Time of Flight Mass Spectrometry analysis (MALDI-TOF-MS) for genotyping. The specific procedure commenced with the design of site-specific PCR primers and extension primers based on locus information. The primer information of these SNPs was listed in Supplementary Table S1. The DNA template containing the SNP site region was then amplified using PCR technology. The resulting PCR products were treated with SAP enzyme to remove excess dNTPs. Subsequently, single-nucleotide extension was performed simultaneously at the target site. The site-specific extension primer extends by one base and terminates at the mutation site. Depending on the mutation type, the extension primer incorporates different dNTPs, leading to molecular weight differences. After purification of the extension products through resin, they were spotted onto a target plate. Finally, a mass spectrometer was employed to detect the molecular weight differences among various extension products. Data analysis enables the determination of the specific genotype for each mutation site.

### 2.5 Statistical analysis

The statistical analysis was conducted using IBM SPSS Statistics for Windows, version 26.0 (IBM Corp., Armonk, N.Y., United States of America), R software (version 4.3.2) and RStudio software. Figures are generated by GraphPad Prism (version 8.0.1 for Windows, San Diego, California United States of America) and RStudio software. During the retrospective data collection phase, we meticulously documented any instances of missing data, including their reasons, to maintain transparency and facilitate accurate interpretation. Variables with a missing rate exceeding 15% were considered for deletion of missing values. The Kolmogorov Smirnov test (K-S test) was used to determine the normal distribution of the data. The Bartlett test or Levene test was used to test the homogeneity of variances. The chi-square test was used to detect differences in qualitative data like sex, age compositions, administrations, and drug interactions between different severe DILI subgroups. Student’s t-test or Mann-Whitney *U* test was used to compare the quantitative data such as MTX dose, baseline biochemical parameters between different severe DILI subgroups. The stepwise regression analysis (backward method) was used to select the potential predictors. Furthermore, the binary logistic regression analysis was performed to analyze the risk/protective factors and their odd ratio for moderate/severe DILI. A nomogram was constructed based on independent factors to visualize predictions for complex models.

Finally, the efficiency and clinical significance of the predictive model was evaluated. All R packages used in this analysis were cited as follows: pROC (Robin et al., 2011), PPROC (Keilwagen et al., 2014), rms (Harrell F. E., 2023), rmda (Brown M, 2018), and MASS (Venables, W. N., 2002). Receiver operating characteristics (ROC) curves from pROC and precision and recall (PR) curves from PRROC were used to explore the prediction performance of the united predictors. Besides, a calibration curve from the rms was used to figure out the optimal alignment between predicted and observed severities. Decision curve analysis (DCA) curve from the rmda was used to evaluate the clinical benefit of the predictive model. MASS was used for establishment of stepwise regression. Values are expressed as mean ± Standard Error of Mean, median (interquartile range) or N (%). A two-tailed *p* < 0.05 was considered statistically significant.

## 3 Result

### 3.1 Patient characteristics and clinical effect on moderate/severe DILI

**FIGURE 1:**
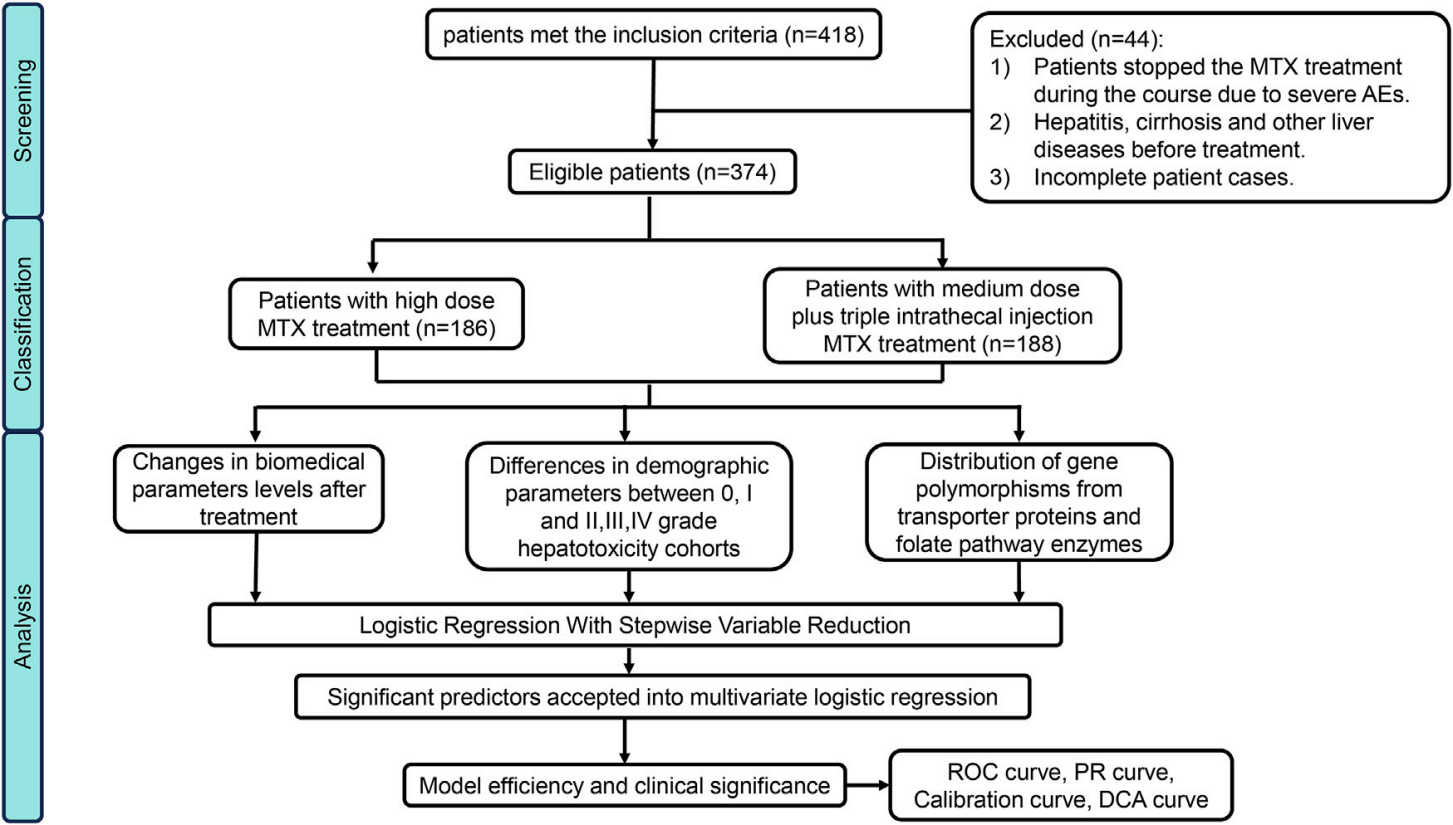
Flowchart of patient inclusion and statistical analysis process.

In this cohort, 418 patients initially met the inclusion criteria, however, 44 individuals were excluded based on our predefined criteria, some of them were unable to tolerate the severe adverse effects including bone marrow suppression, gastric ulcer, mucosal hemorrhage and acute kidney injury and thus changed the treatment regime. Details were as illustrated in the flowchart presented in Figure 1. Ultimately, 374 eligible patients were enrolled in this study (Male: 228; Female: 146). Among them, 186 patients accepted HD-MTX (8–12 g/m^2^) treatment while 188 patients received medium-dose methotrexate (MD-MTX) (1∼8 g/m^2^) in combination with IT-MTX (triple Intrathecal injection). Baseline demographic and pharmaceutical parameters between non/mild and moderate/severe DILI are shown in Table 1. In terms of demographic characteristics, significant differences in sex distribution were observed between the two cohorts: the proportion of male patients (64%) was higher than female patients (36%) in the none/mild DILI cohort (*p* = 0.018), while it was nearly equal in moderate/severe DILI cohort. We distributed ages into for four groups (1–18 years; 19–35 years; 36–50 years; >50 years). Although the composition ratios of four age groups did not exhibit significant differences between the two cohorts, it was evident that children and adolescents constituted the largest age composition in both cohorts (moderate/severe DILI cohort: 73.2%; none/mild DILI cohort: 57.4%). Patients receiving MTX treatment were diagnosed with OS, ALL, NHL and a small portion of other malignant neoplastic disease (Langerhans cell histiocytosis). Moreover, the compositions in the two DILI cohorts differed significantly (*p* < 0.001): In the moderate/severe DILI cohort, majority of patients were treated for OS (70%), while in the none/mild DILI cohort, 162 patients (53.5%) were treated for ALL.

**TABLE 1:** Patient demographic and pharmaceutical characteristics between DILI Grade≥2 and Grade<2 cohorts.

Characteristics	Levels	Moderate/severe DILI		p*
		Yes (N = 71)	No (N = 303)	
Demography				
Sex	Male	34 (47.9%)	194 (64%)	0.018
	Female	37 (52.1%)	109 (36%)	
Age (years)	0-18	52 (73.2%)	174 (57.4%)	0.068
	19-35	11 (15.5%)	56 (18.5%)	
	36-50	5 (7%)	40 (13.2%)	
	>50	3 (4.2%)	33 (10.9%)	
BMI (kg/m^2^)		18.7 (15.6–22.0)	19.0 (16.0–22.9)	0.732
Hospitalizations		5.0 (3.0–11.0)	6.0 (3.0–9.0)	0.996
diagnosis	Acute leukemia	16 (22.9%)	162 (53.5%)	<0.001
	Osteosarcoma	49 (70%)	70 (23.1%)	
	Lymphoma	3 (4.3%)	67 (22.1%)	
	Others	2 (2.9%)	4 (1.3%)	
Treatment				
Administration	HD-MTX	51 (71.8%)	129 (42.6%)	<0.001
	MD-MTX + IT-MTX	20 (28.2%)	174 (57.4%)	
MTX dose (g/d)	Median (IQR)	13.0 (5.5–15.0)	3.2 (1.8–7.3)	<0.001
Concomitant drug use (yes)	PPI	134 (44.2%)	29 (40.8%)	0.701
	NSAIDS	181 (59.7%)	48 (67.6%)	0.276
	SMZ	116 (38.3%)	27 (38%)	1.000
	PPI/NSAIDS/SMZ	235 (77.6%)	54 (76.1%)	0.909
C24hMTX		23 ± 5.9	7 ± 1.0	0.013
C48hMTX		0.2 (0.1–0.6)	0.2 (0.1–0.5)	0.604
C72hMTX		0.2 (0.1–0.6)	0.2 (0.1–0.5)	0.719
Delay in MTX elimination (24 h)	Yes	24 (33.8%)	34 (11.2%)	<0.001
Delay in MTX elimination (48 h)	Yes	7 (9.9%)	24 (7.9%)	0.769
Delay in MTX elimination (72 h)	Yes	9 (12.7%)	55 (18.2%)	0.354

The value was presented as N (%) or Median (interquartile range); Abbreviations: BMI, body mass index; HD-MTX, high dose methotrexate; MD-MTX + IT-MTX, medium dose methotrexate with intrathecal methotrexate; PPI, proton pump inhibitor; NSAIDS, Non-Steroidal anti-inflammatory drugs; SMZ, Sulfamethoxazole. Yes in monotherapy means combination with PPI, NSAIDS, and/or sulfonamides. *The *p*-value less than 0.05 was considered significant. Variables described by Median (IQR) were analyzed by Mann Whitney test. Variables described by Mean (±Standard Error of Mean) were analyzed by Welch Two Sample *t*-test. Variables represented by n (%) were analyzed by either Chi-square test or Fisher’s exact test.

Concerning pharmaceutical characteristics, during the treatment duration of MTX for OS, ALL, and NHL, two administration methods were employed: HD-MTX administrated as IV and IV + IT. Both methods led to varying degrees of liver function impairment (*p* < 0.001). Moderate/severe DILI was more prevalent in patients treated with HD-MTX receiving IV alone compared to those with HD-MTX receiving IV + IT (71.8% vs. 28.2%). Additionally, the MTX dose, which depended on malignant neoplastic disease and body surface area, differed significantly between the two groups (*p* < 0.001). In the moderate/severe DILI subgroup, the medium dose was 13 g/time (interquartile range, 5.5-15.0), while it was 3.2 g/time (interquartile range, 1.8-7.3) in the none/mild DILI subgroup. However, concomitant use of PPI like Omeprazole, NSAIDS like Ibuprofen, and SMZ as well as their different combinations showed no statistical difference in the two cohorts. Notably, at the 24-h clearance delay, 33.8% of patients with moderate/severe DILI exhibited the delay compared to 11.2% in the non-DILI group (*p* = 0.001) while there were no significant differences at 48-h and 72-h clearance delay in the two cohorts. The median (interquartile range, IQR) of 24h MTX concentration in the moderate/severe DILI subgroup was higher than that in the none/mild DILI cohort (1.8 (0.3–21.0): 1.1 (0.3–7.7)) though the difference wasn’t significant (*p* = 0.306).

### 3.2 Effects of differential therapeutic regimens on dose, C_24h_, C/D of MTX

**FIGURE 2:**
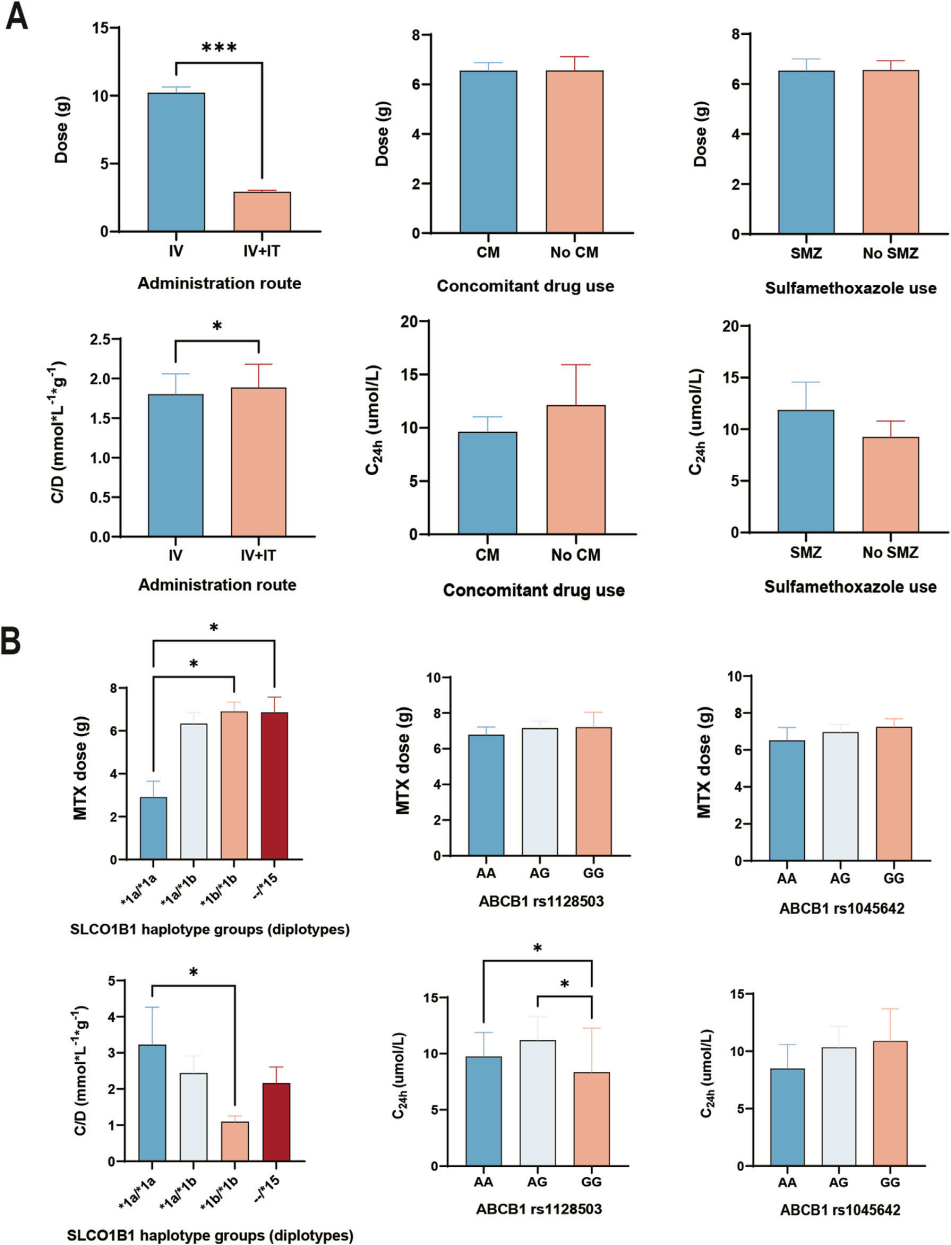
Effect of differential therapeutic regimens and genetic factors on MTX pharmacokinetic parameters. **(A)** Administration route and concomitant drug use associated with MTX pharmacokinetic parameters. **(B)** Genetic effect of genotypes and haplotypes on MTX pharmacokinetic parameters. Analyzed by Kruskal–Wallis test or Mann-Whitney *U* test. IV, HD-MTX using by intravenous injection. IV + IT, HD-MTX using by intravenous and triple intrathecal injection route. CM, concomitant drug use (proton pump inhibitor + non-steroidal anti-inflammatory drugs). SMZ, Sulfamethoxazole. --/*15 means at least one *15 (*1a/*15, *1b/*15, *5/*15).

According to the results of normality presented in Supplementary Table S2, most of the variables were found to be non-normally distributed. This indicates that conducting linear regression analysis on MTX concentration would be inaccurate. However, given the significant influence of C_24h_ on the severity of DILI, we also performed univariate analysis to explore the initial clinical factors related to dose, C_24h_ and C/D which presented in Figure 2A. The Intravenous dose from patients with HD-MTX using IV alone was significantly higher than those who were using IV + IT route (*p* < 0.001). However, there were no significance differences in concomitant drug use groups (CM vs. No CM: *p* = 0.9135) and sulfamethoxazole use groups (SMZ vs. No SMZ: *p* = 0.9499). Dose-adjusted concentration (C/D) refers to a pharmacokinetic metric that adjusts a drug’s concentration in the plasma relative to the dose administered (Tveit et al., 2024). We utilized C/D due to the uneven distribution of dose in the administration route. We found significantly higher C/D levels in HD-MTX when administered via intravenous and intrathecal routes (IV + IT) compared to intravenous (IV) alone (IV + IT vs. IV: *p* = 0.0328). However, there were no significant differences in C_24h_ levels among groups with concomitant drug use (*p* = 0.8919) or sulfamethoxazole use (*p* = 0.2794).

### 3.3 Pharmacogenomic association of MTX transporters and folate pathway with moderate/severe DILI

We genotyped 25 SNPs from 18 genes. The genotype frequencies were calculated and the minor allele frequency was compared with that of Southern Han Chinese (CHS) from 1,000 genomes projects (Table 2). As a result, allele frequencies of all the SNPs were similar to those in the 1,000 genomes projects. We observed no recessive allele mutations or homozygous variance in *GSTP1* rs1695, and *SHMT1* rs1979277 polymorphisms (Table 2). Besides, *SLCO1B1* rs4149081 (*p* = 0.0172) deviated under Hardy-Weinberg Equilibrium test. Thus, these three SNPs were excluded from the subsequent analysis.

**TABLE 2:** Characteristics of targeted SNPs from MTX transporters and folate pathways.

Gene	SNP	Genotype frequency			Allele frequency		Ref^a^	HWE (p*)
		wt	het	var	p	q		
ABCB1	rs1045642	55 (14.8%)	170 (45.7%)	147 (39.5%)	0.3763	0.6237	0.6022	0.8777
	rs1128503	147 (39.5%)	181 (48.4%)	44 (11.8%)	0.6384	0.3616	0.373	0.5760
SLC19A1	rs1051266	87 (23.4%)	182 (48.9%)	103 (27.7%)	0.4785	0.5215	0.5285	0.9304
ARID5B	rs10994982	128 (34.4%)	170 (45.7%)	74 (19.9%)	0.5726	0.4274	0.499	0.4410
GGH	rs11545078	314 (84.4%)	56 (15.1%)	2 (0.54%)	0.9194	0.0806	0.0873	0.9578
	rs3758149	246 (65.8%)	112 (29.9%)	14 (3.7%)	0.8118	0.1882	0.2192	0.9344
ABCG2	rs12505410	147 (39.5%)	177 (47.3%)	48 (12.9%)	0.6331	0.3669	0.3194	0.8904
	rs2231142	166 (44.7%)	172 (46.4%)	33 (8.9%)	0.6792	0.3208	0.2907	0.4685
FPGS	rs1544105	28 (7.53%)	152 (40.9%)	192 (51.6%)	0.2796	0.7204	0.6905	0.9624
GSTP1	rs1695	372 (100.0%)	—	—	1	0	0.1786	—
MTHFR	rs1801131	234 (62.9%)	118 (31.7%)	20 (5.4%)	0.7876	0.2124	0.2192	0.6073
	rs1801133	174 (46.8%)	164 (44.1%)	34 (9.1%)	0.6882	0.3118	0.2956	0.8711
MTRR	rs1801394	209 (56.2%)	139 (37.4%)	24 (6.5%)	0.7487	0.2513	0.2629	0.9896
MTHFD1	rs2236225	225 (60.5%)	129 (34.7%)	16 (4.3%)	0.7824	0.2176	0.1984	0.8811
MTR	rs3768142	59 (15.9%)	174 (46.8%)	139 (37.4%)	0.3925	0.6075	0.5734	0.9335
SHMT1	rs1979277	302 (86.0%)	49 (14.0%)	—	0.9544	0.094	0.0605	—
SLCO1B1	rs2306283	14 (3.8%)	129 (34.9%)	227 (61.4%)	0.2122	0.7878	0.7619	0.7113
	rs4149056	302 (81.2%)	66 (17.7%)	4 (1.1%)	0.9005	0.0995	0.123	0.9830
	rs4149081	91 (24.7%)	211 (57.2%)	67 (18.2%)	0.5325	0.4675	0.45	0.0172
DHFR	rs442767	52 (14.1%)	155 (41.9%)	163 (44.1%)	0.35	0.65	0.5853	0.3131
ABCC2	rs717620	235 (63.2%)	124 (33.3%)	13 (3.5%)	0.7984	0.2016	0.2173	0.7917
	rs3740065	153 (41.1%)	170 (45.7%)	49 (13.2%)	0.6398	0.3602	0.3095	0.9864
ABCC1	rs246240	140 (37.6%)	189 (50.8%)	43 (11.6%)	0.6304	0.3696	0.37	0.2200
DROSHA	rs639174	32 (8.65%)	154 (41.6%)	184 (49.7%)	0.2946	0.7054	0.6944	0.9994
ABCC4	rs7317112	172 (46.4%)	162 (43.7%)	37 (10.0%)	0.6819	0.3181	0.3661	0.9912

wt, Homozygous wild-type patient; het, heterozygous variant patient; var, homozygous variant patient. The value was presented as N (%). p, frequency of wild type allele; q, frequency of variant allele; a, ref represents the MAF, value of CHS, from 1000 Genomes Project; *p-value of Hardy-Weinberg equilibrium analysis, *p* > 0.05 was considered to meet the requirements for inclusion.

From the univariable analysis of the correlation between genetic polymorphisms and outcome in Supplementary Table S3, two SNPs (*ABCB1* rs1128503, *ABCB1* rs1045642) were significantly associated with moderate/severe DILI. The risk of moderate/severe DILI in patients with *ABCB1* rs1128503 GG + GA was significantly higher than that with *ABCB1* rs1128503 AA (OR (95% CI): 1.88 (1.08-3.38); *p* = 0.028). The risk of moderate/severe DILI in patients with *ABCB1* rs1045642 GG + GA was significantly higher than that with AA (OR (95% CI): 2.63 (1.01-6.86, *p* = 0.048) patients.

### 3.4 Effects of ABCB1 genotypes and SLCO1B1 haplotypes on dose, C_24_, C/D of MTX

Due to the strong linkage disequilibrium of the *SLCO1B1* gene, where the inheritance of each SNP cannot be explored independently, we conducted haplotype analysis on *SLCO1B1*, including *1a (wild type at all loci), *1b (rs2306283 G allele), *5 (rs4149056 C allele), and *15 (rs2306283 G allele and rs4149056 C allele). In this study, the mutant frequency of *1b (70% in Non/mild DILI subgroup and 65% in Moderate/severe DILI subgroup) was much higher than that of *1a and *15. However, there were no mutations in the *5 haplotype (Table 3). Among the haplotype groups, patients with the *15 haplotype exhibited no significant difference for 24h MTX clearance and severity of DILI compared to patients with *1a/*1a, *1b/*1b, and *1a/*1b.

**TABLE 3:** Association between SLCO1B1 haplotype and severity of DILI and MTX clearance.

Characteristics	Non/mild DILI (N = 606)	Moderate/severe DILI (N = 142)	*p* ^*a*^	No delay in clearance (N = 574)	Delay in clearance (N = 174)	*p* ^*a*^
Haplotype			0.163			0.701
*1a	119 (20%)	38 (27%)		132 (21.2%)	25 (21.6%)	
*1b	416 (70%)	93 (65%)		432 (69.2%)	77 (66.4%)	
*5	0	0		0	0	
*15	63 (11%)	11 (7.7%)		60 (9.6%)	14 (12.1%)	
Haplotype groups			0.247			0.459
*1a/*1b, *1a/*1a, *1b/*1b	239 (80%)	61 (86%)		255 (81.7%)	45 (77.6%)	
--/*15	60 (20%)	10 (14%)		57 (18.3%)	13 (22.4%)	

The value was presented as N (%), a, analyzed by either Chi-square test or Fisher’s exact test. --/*15 means at least one *15 (*1a/*15, *1b/*15, *5/*15).

As shown in Figure 2B, the dose of HD-MTX from patients with *1a/*1a haplotype was significantly lower than patients with *1b/*1b and --/*15 (H = 9.069, *1a/*1a vs. *1b/*1b: *p* = 0.0177; *1a/*1a vs. --/*15: *p* = 0.0383). However, the dose of HD-MTX from patients with different genotypes showed no significant difference in *ABCB1* rs1128503 (H = 1.517, *p* = 0.4683) and *ABCB1* rs1045642 (H = 0.9494, *p* = 0.6221). Regarding dose adjusted concentration, patients with *1a/*1a had significantly higher concentrations than those with *1b/*1b (H = 9.705, *1a/*1a vs. *1b/*1b: *p* = 0.0196). For MTX concentration at 24h, patients with GG genotype in ABCB1 rs1128503 had significantly lower concentrations than those with AA and GA (H = 9.183, AA vs. GG: *p* = 0.0122; AG vs. GG: *p* = 0.0128). However, there was no significant differences of C_24h_ among each genotype in *ABCB1* rs1045642 (H = 1.259, *p* = 0.5327).

### 3.5 Building predictive model with genetic and clinical factors for moderate/severe DILI

Based on genetic polymorphisms and demography, pharmacokinetics, baseline biochemical parameters of enrolled patients, 50 factors were included for stepwise regression analysis (backward). The following factors were selected into binary logistic regression model: sex, MTX dose, MTX concentration at 24h, serum creatinine, *ABCB1* rs1128503, and *SLCO1B1* haplotype groups. The result of binary logistic regression model is shown in Table 4. According to the final logistic regression model, five independent risk factors for developing moderate/severe DILI were identified: sex (female vs. male: OR (95%Cl): 2.184 (1.15-4.14); *p* = 0.017); MTX dose (OR (95%Cl): 1.205 (1.14-1.28); *p* < 0.001); *ABCB1* rs1128503 polymorphism (GG + GA vs. AA: OR (95%Cl): 2.121 (1.11-4.05); *p* = 0.023); *SLCO1B1* *1b/*1b (*1b/*1b vs. *1a/*1a: OR (95%Cl): 0.187 (0.04-0.91); *p* = 0.038); *SLCO1B1* *1b/*15 (*1b/*15 vs. *1a/*1a: OR (95%Cl): 0.106 (0.02-0.68); *p* = 0.018).

**TABLE 4:** Binary logistic regression analysis of moderate and severe DILI predictors.

Predictor	Estimate	95% CI lower	95% CI upper	SE	Z	*p*	OR
Intercept	−2.12459	−3.83732	−0.41186	0.87386	−2.431	0.015	0.119
Sex							
Female vs. Male	0.78127	0.14191	1.42062	0.32621	2.395	0.017	2.184
MTX dose (g)	0.18660	0.12879	0.24441	0.02950	6.326	<0.001	1.205
C_24h_MTX (μmol/L)	0.00369	−0.00580	0.01318	0.00484	0.762	0.446	1.004
Cr (μmol/L)	−0.01461	−0.03346	0.00423	0.00961	−1.520	0.128	0.985
ABCB1.rs1128503							
GG + GA vs. AA	0.75206	0.10507	1.39905	0.33010	2.278	0.023	2.121
SLCO1B1 haplotypes							
*1b/*1b vs. *1a/*1a	−1.67769	−3.26529	−0.09009	0.81002	−2.071	0.038	0.187
*1a/*1b vs. *1a/*1a	−0.76200	−2.26238	0.73839	0.76552	−0.995	0.320	0.467
*1b/*15 vs. *1a/*1a	−2.24372	−4.09526	−0.39218	0.94468	−2.375	0.018	0.106
*1a/*15 vs. *1a/*1a	−1.62841	−3.64182	0.38501	1.02727	−1.585	0.113	0.196
*15/*15 vs. *1a/*1a	−1.33123	−4.27360	1.61114	1.50124	−0.887	0.375	0.264

^a^ Estimates represent the log odds of “moderate/severe DILI” vs. “non/mild DILI”. OR, for MTX, dose, C_24h_MTX, and Cr means risk effect size of 1-unit increased in the values of each of these variables.

**FIGURE 3:**
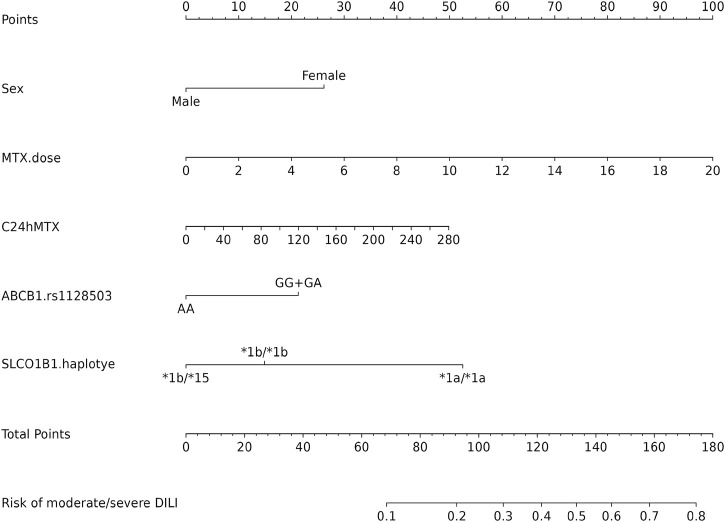
Development of a nomogram for predicting risk of DILI (grade≥2). The nomogram included sex, MTX dose, C_24h_ MTX, *ABCB1* rs1128503, *SLCO1B1* haplotypes. The nomogram summed the scores for each scale and variable. The total score on each scale indicated the risk of moderate/severe DILI.

A nomogram comprised of these four independent factors and MTX concentration at 24 h was created to predict the probability of the risk of moderate/severe DILI (Figure 3). Scores were allocated to each variable based on the demographic and clinical characteristics of the patients. The overall score was then calculated by summing these individual scores. After that, the nomogram provided us with an equation to predict the probability of outcome based on total scores:

Risk of moderate/severe DILI = −5.15 * 10^−7^ * points ^3 + 0.000204917 * points ^2 - 0.018926805 * points +0.601399956.

### 3.6 Validating the predictive model for risk of moderate/severe DILI

**FIGURE 4:**
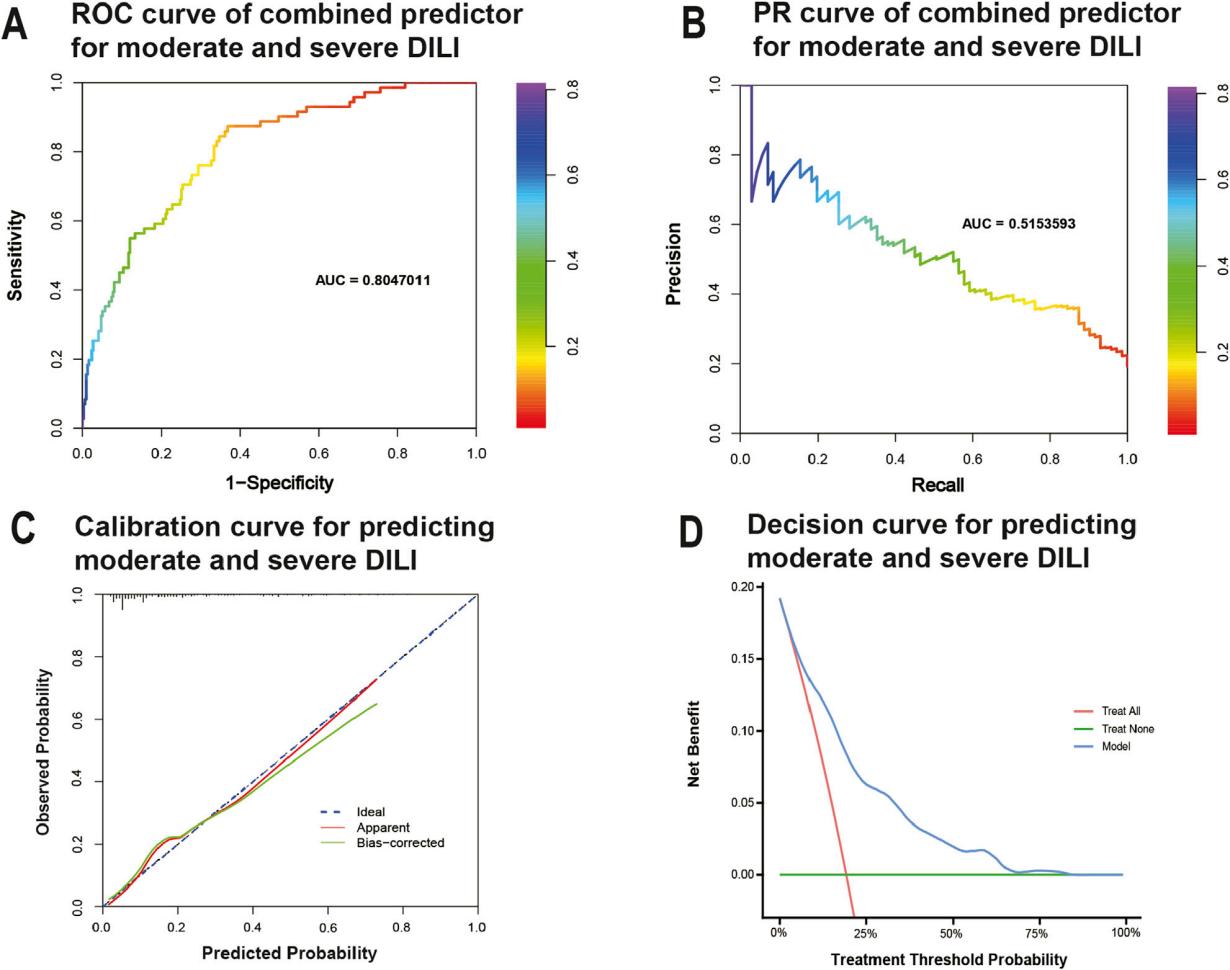
Evaluation of practical clinical application value of the model. **(A)** Receiver-operating characteristic curve for predicting moderate/severe DILI. **(B)** precision–recall curve for predicting moderate/severe DILI. **(C)** Calibration curves for predicting moderate/severe DILI. Data on predicted and observed probability were plotted on the x- and *y*-axis, respectively. The ideal 45-degree line (referred to as the “deadline”) serves as a reference standard. The “apparent” line reflects the degree of fit between predicted values and actual values, while the solid line after bias correction (Bias-corrected line) illustrates the fit between post-correction predicted values and actual values. If the Bias-corrected line or the Apparent line is close to the ideal line, it indicates better consistency between predicted and actual values. **(D)** Decision curves predicting moderate/severe DILI. The *x*-axis represents threshold probabilities and the *y*-axis measures the net benefit calculated by adding true positives and subtracting false positives. It helps assess whether a model improves decision-making for patient outcomes by balancing the benefits of true positives against the harms of false positives.

The Area Under the Curve (AUC) of the ROC curve (AUROC) in Figure 4A and the Precision-Recall curve (AUPRC) in Figure 4B were used to evaluate the predictive accuracy and efficiency of the model. The AUROC was 0.805 (95% CI, 0.750 - 0.859) based on false positive rate and true positive rate, while the AUPRC was 0.515 based on precision and recall parameters. The calibration plots for the nomogram model indicated an optimal alignment between predicted and observed severities. As illustrated in Figure 4C, the bias-corrected line and apparent line approached the ideal line, indicating a high level of consistency in predicted and observed severities. Furthermore, Decision Curve Analysis (DCA) revealed that the predictive model provided significant net benefits across a range of threshold probabilities at various time points, underscoring its potential clinical utility (Figure 4D).

## 4 Discussion

This retrospective longitudinal study, coupled with genetic testing, provided a thorough examination of the hepatotoxic effects induced by HD-MTX incorporating clinical and biochemical parameters, MTX concentrations at multiple time points, drug interactions as well as MTX administration methods. The contributions of this study to the field are outlined as follows: 1) Establishment of a novel warning model presented by a logistic regression nomogram, emphasizing its incorporation of clinical parameters, MTX genotypic mutations, and MTX dosage; 2) Identification of possible independent risk factors, specifically *ABCB1* rs1128503, and protective factors, such as *SLCO1B1* haplotypes, for moderate/severe DILI in Han Chinese population with ALL, NHL, OS; 3) Discovery of significantly higher prevalence of higher dose adjusted concentration in patients with HD-MTX using intravenous plus triple intrathecal injection route than those using intravenous injection alone.

Numerous studies have aimed to establish predictive models for MTX post-treatment toxicity (Kawakatsu et al., 2019; Li et al., 2019; Onishi et al., 2020; Zobeck et al., 2023). This involves a multidisciplinary integration process, with one aspect being the establishment of population pharmacokinetic (pop PK) model (Shi et al., 2020; Chen et al., 2023; Jung et al., 2023). Kawakatsu et al. proposed that the estimated glomerular filtration rate and cycle number were independent predictors of MTX clearance and they employed the final pop PK model to generate anticipated exposures for different MTX dosing regimens (Kawakatsu et al., 2019). The assessment of MTX exposure, manifested as blood concentration or drug-time curve, is directly associated with toxicity. Exploring the levels and factors influencing them holds significant relevance. However, it's important to note that this approach essentially serves as a retrospective salvage method, as the risk of toxicity often accompanies drug usage. Another commonly employed strategy is the use of genomics, incorporating genetic profiling as a predictive factor into risk prediction models (He et al., 2021; Lu et al., 2021; Membrive-Jiménez et al., 2023). The predictive model in this area can be presented as traditional regression model, visual nomogram, or machine learning based model. A recent study using Bayesian hierarchical ordinal regression models for prolonged MTX clearance suggested that older ages, B-ALL type, minor alleles of *SLC19A1* rs2838958 and *ABCC4* rs7317112 were associated with high risk for prolonged clearance (Zobeck et al., 2023). This approach is prospective and provides detectable results before treatment initiation. However, the selection of gene polymorphisms related to MTX varies widely among individuals and populations due to different mutation frequencies, adding complexity to the interpretation of genetic factors influencing MTX pharmacokinetics. Above all, the occurrence of potential toxicity accompanied with post-treatment blood drug concentration monitoring and the racial differences in the selection of pre-treatment genetic testing are also unsolved issues for present study and area in developing predictive models for clinical toxicity.

The initial phase involves placing increased emphasis on pre-treatment genetic markers and drug transporters in the field of MTX toxicity. It is crucial to underscore that the majority of predictions regarding clinical toxicity induced by HD-MTX come from therapeutic drug monitoring (TDM), which is indeed efficient but operates post-treatment. A Chinese guideline for HD-MTX treatment strongly recommends TDM during therapy (Song et al., 2022). However, genetic testing is only weakly recommended in this guideline. Moreover, existing clinical practices primarily focus on genes associated with the folate pathway, such as *MTHFR* and *DHFR* (Erčulj et al., 2012). Although some research indicated a correlation between *MTHFR* gene polymorphisms and MTX blood concentrations (Suthandiram et al., 2014; Choi et al., 2017; Wang et al., 2017), it is crucial to acknowledge that the primary impact of *MTHFR* and *DHFR* mostly reflect the antifolate efficacy of MTX (Jabeen et al., 2015). In the context of HD-MTX, where hepatotoxicity is a critical limiting factor, investigating transporter genes such as *SLC19A1*, *ABCB1*, and *SLCO1B1* becomes more important. Individuals with hidden mutations in these transporter genes may experience a weakened transport efficiency of MTX, potentially increasing the risk of hepatotoxicity (Cheng et al., 2021). Pre-treatment testing for transporter genotypes could offer a means to better tailor treatment plans, mitigating the risk of moderate and severe DILI.

Secondly, formulating a potential solution for selecting SNPs into a predictive model across diverse ethical considerations is a challenging task. This may necessitate collaboration among various countries and medical centers. While there is ample evidence supporting the existence of numerous predictive models incorporating pharmacogenetic analysis from different regions, the challenge lies in the lack of compatible methodological standards. The varied results of significant SNPs from distinct predictive models cannot be easily amalgamated. Instead, a unified and rigorously methodology-based model is required for adoption across all medical centers. In present predictive model based on the population from south China, we identified the *ABCB1* rs1128503 and *SLCO1B1* haplotypes (consist of rs2306283 and rs4149056) as independent factors for moderate/severe DILI. However, the minor allele frequency of these SNPs shows regional disparity: Data from the 1000 Genomes project indicated that the frequency of *ABCB1* rs1128503 in East Asians was lower than Europeans and Americans (rs1128503 SNP - Population genetics - Homo_sapiens - Ensembl genome browser 110, 2023). Whereas the frequency of *SLCO1B1* rs2306283 in East Asians was higher (rs2306283 SNP - Population genetics - Homo_sapiens - Ensembl genome browser 110, 2023), which proved the effect of genotypes and following predictive model within genotypes should be discussed separately.

Adenosine triphosphate (ATP)-binding cassette (ABC) superfamily, *ABCB1* and solute carrier organic anion transporter family member 1B1, *SLCO1B1* show different physiological functional directions in the liver. For one hand, *ABCB1* located on the bile canalicular membrane of hepatocytes to facilitate the drugs eliminated off the liver (Ho and Kim, 2005; Giacomini et al., 2010). Variant polymorphism in *ABCB1* may alter different functions of P-glycoprotein (P-gp): overexpression of P-gp leads to enhanced elimination of MTX through the bile excretion; While silent *ABCB1* expression may contribute to the intracellular accumulation of MTX, thereby associated toxicity arise (Kato et al., 2012). Studies have shown that the rare allele of *ABCB1* rs1045642 alone or in combination with the rare alleles of rs1128503 reduced the P-glycoprotein activity and increased the risk of drug toxicity subsequently (Kimchi-Sarfaty et al., 2007; Gong et al., 2021), which is also accordance with our results. For other hand, *SLCO1B1*, encoding the hepatic uptake transporter *OATP1B1*, is crucial for drugs like statins and MTX (Niemi et al., 2011). Interestingly, genetic polymorphisms seem more complicated in *SLCO1B1* which includes various SNPs and haplotypes like *5, *15, *14, and *17 (Ramsey et al., 2023). These SNPs and haplotypes exhibited different activities of *OATP1B1* in liver accounting for individual differences in hepatocellular uptake, hepatic clearance, and drug systemic exposure (Wagner et al., 2019; Wagner et al., 2020). The haplotype *1b (rs2306283) is associated with increased activity of *OATP1B1*, while haplotype *5 (rs4149056) and *15 (rs23062836+ rs4149056) is associated with decreased activity (Mwinyi et al., 2004; Li et al., 2012). Our results indicated that patients with *1b/*1b and *1b/*15 genotypes may be protective against moderate/severe drug-induced liver injury (DILI). This could be explained by the *1b mutation enhancing the efficiency of hepatic uptake transporters, leading to increased elimination of methotrexate (MTX) and thereby reducing MTX concentration and alleviating DILI. This is supported by a study by Maeda et al., which reported that patients carrying the SLCO1B1 *1b allele had a lower prevalence of drug-induced adverse reactions such as increased AST levels and anemia (Maeda et al., 2017). Except the *1a, *1b, *5, and *15 in this study, other haplotype like *14 also has great impact on hepatic uptake. Studies have shown that *14 (rs2306083 + rs11045819) was associated with the increased *OATP1B1* function and MTX clearance (Ramsey et al., 2012; Nies et al., 2013). Therefore, the more predictive value of haplotypes in SLCO1B1 variants for DILI need to be further explored in the future.

Except for genetic polymorphism, different concomitant drug use showed different specific mechanism toward MTX therapeutic toxicity. On one hand, the effect of NSAIDS and PPIs interaction on MTX is more closed to the competition of drug transporter and the following MTX pharmacokinetics. Previous reports suggest that NSAIDs may increase MTX concentration by diminishing its renal tubular excretion, possibly through competition for secretion via the renal organic anion transporter (Kawase et al., 2016). Other data propose that PPIs competing for breast cancer–resistant protein efflux transporters (BCRP) in the hepatobiliary and renal proximal tubule may contribute to a reduction in MTX elimination (Bezabeh et al., 2012). On the other hand, the combination of SMZ, which acts on dihydrofolate synthase, and MTX, which selectively inhibits dihydrofolate reductase, results in a dual blockade of cellular folate metabolism, leading to subsequent cellular toxicity (Jafari et al., 2023; Zhu et al., 2023). In present study, we did not find a statistically significant association between concomitant drugs use and the occurrence of moderate/severe DILI. This may be explained by the fact that combination therapy can influence methotrexate (MTX) exposure, even though clinicians may implement liver protection measures when combining medications, aiming to achieve a balance between benefits and risks. However, due to the initial influence of co-therapy on MTX, it is still necessary to maintain pharmacovigilance when applying combination therapy.

Significant differences in gender, drug dosage, and administration methods were observed between the two groups of patients with and without severe DILI. These differences are nonnegligible for interpreting the intra-patient exposure to MTX and potential toxic reactions. Firstly, we noted a higher proportion of severe DILI in females in this cohort compared to males. This may be related to physiological differences between females and males (Ivan et al., 2023). For instance, females may exhibit distinct physiological characteristics such as body fat content, liver enzyme activity, and hormone levels (Waxman and Holloway, 2009; Taylor et al., 2010; Ferri et al., 2022; Mease et al., 2022), which could impact the pharmacokinetics and pharmacodynamics of MTX. Additionally, female patients might be more susceptible to hormonal changes in certain situations, potentially increasing the risk of DILI (Amacher, 2014). However, these speculations require further in-depth research for validation. A study in Norway supported this result suggesting that lower initial folate concentrations in females compared with males and young female patients were most affected by HD-MTX hepatic toxicity (Holmboe et al., 2012). Secondly, we observed that different administration methods reflected differences in drug dosage, subsequently affecting intra-patient exposure to MTX. Generally, HD-MTX short-term continuous infusion therapy, which involved higher dosage (8–12 g/m^2^), is often associated with adverse reactions such as mucositis, myelosuppression, and DILI (Howard et al., 2016). Conversely, HD-MTX using IV (1∼8 g/m^2^) with IT frequently used for ALL and primary CNS lymphomas is more common to see neurological toxicity (Kwong et al., 2009). Thus, their separate drug exposures lead to the different toxicity reaction. Overall, nomogram provides a visual representation of a statistical model, allowing clinicians to predict the probability of adverse events based on individual patient characteristics. Though we can easily acquire the risk probabilities by calculating the risk score from nomogram, there was limited studies that specifically address risk thresholds derived from nomograms for DILI. These risk probabilities were usually presented as reference in clinical practice. A study by Ji et al. performed the nomogram about risk of DILI related to tuberculosis treatment show that risk models help clinicians identify patients who might need closer monitoring or preventive measures based on their probability scores (Ji et al., 2023). Typically, patients with a 20%–30% probability of moderate/severe DILI might require increased monitoring, whereas higher probabilities (50% or greater) may prompt preemptive interventions.

While the study presents significant findings, it is important to acknowledge its limitations. Firstly, the sample size and demographic composition may limit the generalizability of the results. Further, our study focused on the hepatocellular liver injury presented by ALT and AST, but the cholestatic liver injury and mixed liver injury have not been considered. Since hepatocellular DILI is considered to be most relevant for patient outcomes in DILI, which is more so than DILI with mixed or cholestatic injury pattern (Kaplowitz, 2004). A holistic approach, considering all potential variables influencing DILI and complex DILI type, is essential for accurate risk assessment and management in future clinical practice. Secondly, future research should focus on expanding the predictive model’s applicability to diverse populations and drug regimens as an external validation. Investigating the model’s efficacy in predicting DILI in different ethnic groups is crucial, given the genetic diversity across populations. Thirdly, while backward stepwise regression was utilized for variable selection in our predictive model, which influenced the stability of the selected predictors, and future studies should explore alternative variable selection methods to enhance model robustness. Finally, long-term use of non-high-dose MTX in the treatment of rheumatic diseases is also associated with liver injury, typically manifested as mild elevations in liver enzyme levels (Katchamart et al., 2009; Visser and van der Heijde, 2009; Koutsompina et al., 2021). In the future, expanding research on MTX-induced liver injury could further enhance the accuracy of prediction models. Longitudinal studies examining the long-term outcomes of patients identified as high risk for DILI will also provide valuable insights into the model’s impact on patient care.

## 5 Conclusion

We developed a predictive warning model incorporating *ABCB1* and *SLCO1B1* genetic polymorphisms, baseline clinical characteristics, and MTX pharmacokinetic parameters to anticipate moderate and severe DILI among Chinese patients with HD-MTX treatment at an early stage. Our model suggested that female gender, recessive mutation in ABCB1 rs1128503, and a range of MTX concentration may be risk factors for increased susceptibility to moderate/severe DILI while patients with SLCO1B1 *1b/*1b and *1b/*15 may be more prevalent to avoid it. Significantly, our study provides clinicians a more convenient approach to preventing MTX-induced liver injury at the early stages of patient admission, therefore, offering flexibility in guiding tumor chemotherapy regimens involving HDMTX. Future studies should carry out the deep validation of predictive model and consider larger, longitudinal cohorts and genetic differences across diverse ethnic populations.

## Data Availability

The datasets presented in this article are not readily available because of privacy reasons. Requests to access the datasets should be directed to the corresponding authors.
