# Supplementary material for: Integration of genomics, clinical characteristics and baseline biological profiles to predict the risk of liver injury induced by high-dose methotrexate
Source: Front Pharmacol. 2024 Nov 28;15:1423214. doi: 10.3389/fphar.2024.1423214 (PMC11634619; doi:10.3389/fphar.2024.1423214)
Supplement: Supplementary file 1 [file Table1.DOCX]

***Supplementary material***

**Integration of genomics, clinical characteristics and baseline biological profiles to predict the risk of liver injury induced by high-dose methotrexate**

**Supplementary Table 1.** Primers of candidate SNPs

**Supplementary Table 2.** Baseline biochemical characteristics of patients with HD-MTX from two DILI

**Supplementary Table 3.** Univariate analysis of genetic factors (Logistic regression)

**Supplementary Table 4.** Validation of model predictive performance using clinical samples

**Supplementary Table 5.** TRIPOD checklist

**Table S1** Primers of candidate SNPs

| SNP_ID | 2nd-PCRP | 1st-PCRP |
| --- | --- | --- |
| rs3758149 | ACGTTGGATGTCAGGCGCCTTCTAGAATC | ACGTTGGATGAGTCACCCGTACAAAGCTG |
| rs7317112 | ACGTTGGATGTGTTTTTGGTAGAGCCCAGC | ACGTTGGATGAATACAGAGACAGCTGCCG |
| rs10994982 | ACGTTGGATGAGCACATCTGAGGTACAGAG | ACGTTGGATGCGGACCATCATGGTCTTTTA |
| rs1801394 | ACGTTGGATGGAAAATCCATGTACCACAGC | ACGTTGGATGTATGCTACACAGCAGGGAC |
| rs1128503 | ACGTTGGATGTTTCTCACTCGTCCTGGTAG | ACGTTGGATGTTCCCACAGCCACTGTTTC |
| rs1801133 | ACGTTGGATGCACTTGAAGGAGAAGGTGTC | ACGTTGGATGCTTCACAAAGCGGAAGAATG |
| rs12505410 | ACGTTGGATGCCTTGGCACCTTAAATGAAC | ACGTTGGATGGATTCAAACTTGGCCTCTG |
| rs1695 | ACGTTGGATGGCAGATGCTCACATAGTTGG | ACGTTGGATGTGGACATGGTGAATGACGG |
| rs11545078 | ACGTTGGATGCTTTCACTGCTGATTAGTGG | ACGTTGGATGAGTGAAGTTCAGCGGCATTG |
| rs246240 | ACGTTGGATGCAGGGCAAACAAATCACCAG | ACGTTGGATGAGTGCACCCAGCTGTTATC |
| rs717620 | ACGTTGGATGCAGCATGATTCCTGGACTG | ACGTTGGATGCTGTTCCACTTTCTTTGATG |
| rs2236225 | ACGTTGGATGCACATGGCAATTCCTCCATC | ACGTTGGATGTAACCTACAAACCCTTCTGG |
| rs1801131 | ACGTTGGATGTCTACCTGAAGAGCAAGTCC | ACGTTGGATGTCTCCCGAGAGGTAAAGAAC |
| rs3740065 | ACGTTGGATGTCTACTCGGGATACTTGAGC | ACGTTGGATGCACCTAGTGGTCAGATACAG |
| rs1544105 | ACGTTGGATGCTGTGGAAGGGCATTCATTC | ACGTTGGATGCCAACTCCCAGAAATCAAGC |
| rs1045642 | ACGTTGGATGCATTGCCTATGGAGACAAC | ACGTTGGATGAAGGCATGTATGTTGGCCTC |
| rs639174 | ACGTTGGATGTCCTGACTTCTTCTAGTCTC | ACGTTGGATGGCAAGACTCCTTCTCAAAG |
| rs4149081 | ACGTTGGATGGAAGAAACTGTGATTCAAGG | ACGTTGGATGTCACATACAAATCCTAGCC |
| rs4149056 | ACGTTGGATGTATGGGAGTCTCCCCTATTC | ACGTTGGATGAATCTGGGTCATACATGTGG |
| rs1979277 | ACGTTGGATGTCCTTTAGAAGTCAGGCAGG | ACGTTGGATGAGTTCAAGGAGAGACTGGC |
| rs1051266 | ACGTTGGATGAGAAGCAGGTGCCCGTGGAA | ACGTTGGATGTGAAGCCGTAGAAGCAAAGG |
| rs3768142 | ACGTTGGATGAGTCTAACTGGAGAAACAC | ACGTTGGATGAGCCAAAGGAGTGTTTGAC |
| rs2231142 | ACGTTGGATGTGATGTTGTGATGGGCACTC | ACGTTGGATGGTCATAGTTGTTGCAAGCCG |
| rs442767 | ACGTTGGATGTGGGTCACCTGCACAGTATT | ACGTTGGATGAACACGGTAGGTGGCTAAAC |
| rs2306283 | ACGTTGGATGACAAGTGGATAAGGTCGATG | ACGTTGGATGGATGTTCTTACAGTTACAGG |

**Table S2** Baseline biochemical characteristics of patients with HD-MTX from Non/mild DILI and moderate/severe DILI subgroups

| **Characteristic** | **Moderate/severe DILI** | | **p^a^** | **W** | **p^b^** |
| --- | --- | --- | --- | --- | --- |
|  | **No (N = 303)** | **Yes (N = 71)** |  |  |  |
| **ALT (U/L)** | 15 (10, 27) | 13 (9, 20) | 0.305 | 0.22298185 | <0.001 |
| **AST (U/L)** | 22 (17, 33) | 18 (16, 27) | 0.402 | 0.31667418 | <0.001 |
| **TB (g/L)** | 66 ± 7 | 68 ± 7 | 0.016 | 0.99659600 | 0.617 |
| **ALB (g/L)** | 39.7 (37.1, 42.8) | 41.7 (38.1, 43.6) | 0.508 | 0.41243678 | <0.001 |
| **GLB (g/L)** | 25.5 (22.5, 28.8) | 26.8 (24.5, 29.8) | 0.185 | 0.98580443 | <0.001 |
| **TBIL (umol/L)** | 7.9 (5.1, 11.1) | 8.5 (5.9, 11.5) | 0.526 | 0.26622983 | <0.001 |
| **DBIL (umol/L)** | 2.90 (2.00, 4.25) | 2.90 (2.25, 3.65) | 0.584 | 0.17498618 | <0.001 |
| **IBIL (umol/L)** | 4.7 (3.1, 6.5) | 5.4 (3.8, 7.5) | 0.188 | 0.49317051 | <0.001 |
| **BUN (umol/L)** | 4.70 (3.80, 5.79) | 4.70 (3.86, 5.53) | 0.218 | 0.07461336 | <0.001 |
| **Cr (umol/L)** | 55 (41, 68) | 51 (43, 61) | 0.031 | 0.67954496 | <0.001 |
| **UA (umol/L)** | 327 (262, 415) | 320 (261, 368) | 0.066 | 0.74062534 | <0.001 |
| **PLT** | 177 (52, 283) | 270 (190, 319) | <0.001 | 0.96101841 | <0.001 |
| **WBC** | 7 (5, 16) | 7 (5, 9) | 0.140 | 0.31913118 | <0.001 |
| **Hb** | 107 (78, 130) | 125 (108, 136) | 0.001 | 0.98069210 | <0.001 |

The value was presented as N (%) or Median (interquartile range); Abbreviations: ALT, alanine aminotransferase; AST, aspartate aminotransferase; TP, total protein; ALB, albumin; GLB, globulin; TBIL, total bilirubin; DBIL, direct bilirubin; IBIL, indirect bilirubin; BUN, Blood Urea Nitrogen; Cr, creatine; UA, urid acid; PLT, blood platelet; WBC, white blood cell; Hb, hemoglobin. **a**, variables described by Median (IQR) were analyzed by Mann Whitney test. Variables described by Mean (±Standard Error of Mean) were analyzed by Welch Two Sample t-test. **b**. Normality tests (Shapiro-Wilk test).

**Table S3** Univariate analysis of genetic factors (Logistic regression)

| **SNP** |  | **Moderate/Severe DILI** | | **OR (univariable)** |
| --- | --- | --- | --- | --- |
|  |  | **No** | **Yes** |  |
| ABCB1 rs1045642 | AA | 50 (90.9) | 5 (9.1) | - |
|  | GG+GA | 251 (79.2) | 66 (20.8) | 2.63 (1.01-6.86, p=0.048) |
| SLC19A1 rs1051266 | TT | 74 (85.1) | 13 (14.9) | - |
|  | CC+TC | 227 (79.6) | 58 (20.4) | 1.45 (0.75-2.80, p=0.263) |
| ARID5B rs10994982 | AA | 100 (78.1) | 28 (21.9) | - |
|  | GG+GA | 201 (82.4) | 43 (17.6) | 0.76 (0.45-1.30, p=0.322) |
| ABCB1 rs1128503 | AA | 127 (86.4) | 20 (13.6) | - |
|  | GG+GA | 174 (77.3) | 51 (22.7) | 1.86 (1.06-3.28, p=0.031) |
| GGH rs11545078 | GG | 255 (81.2) | 59 (18.8) | - |
|  | AA+GA | 46 (79.3) | 12 (20.7) | 1.13 (0.56-2.26, p=0.735) |
| ABCG2 rs12505410 | TT | 119 (81.0) | 28 (19.0) | - |
|  | GG+GT | 182 (80.9) | 43 (19.1) | 1.00 (0.59-1.70, p=0.988) |
| FPGS rs1544105 | CC | 24 (85.7) | 4 (14.3) | - |
|  | TT+TC | 277 (80.5) | 67 (19.5) | 1.45 (0.49-4.32, p=0.504) |
| MTHFR rs1801131 | TT | 195 (83.3) | 39 (16.7) | - |
|  | GG+GT | 106 (76.8) | 32 (23.2) | 1.51 (0.89-2.55, p=0.123) |
| MTHFR rs1801133 | GG | 141 (81.0) | 33 (19.0) | - |
|  | AA+GA | 160 (80.8) | 38 (19.2) | 1.01 (0.60-1.70, p=0.956) |
| MTRR rs1801394 | AA | 171 (81.8) | 38 (18.2) | - |
|  | GG+GA | 130 (79.8) | 33 (20.2) | 1.14 (0.68-1.92, p=0.615) |
| ABCG2 rs2231142 | GG | 133 (80.1) | 33 (19.9) | - |
|  | TT+GT | 167 (81.5) | 38 (18.5) | 0.92 (0.55-1.54, p=0.744) |
| MTHFD1 rs2236225 | GG | 180 (80.0) | 45 (20.0) | - |
|  | AA+AG | 119 (82.1) | 26 (17.9) | 0.87 (0.51-1.49, p=0.622) |
| SLCO1B1 rs2306283 | AA | 11 (78.6) | 3 (21.4) | - |
|  | GG+GA | 288 (80.9) | 68 (19.1) | 0.87 (0.24-3.19, p=0.828) |
| ABCC1 rs246240 | AA | 110 (78.6) | 30 (21.4) | - |
|  | GG+GA | 191 (82.3) | 41 (17.7) | 0.79 (0.47-1.33, p=0.372) |
| ABCC2 rs3740065 | AA | 119 (77.8) | 34 (22.2) | - |
|  | GG+GA | 182 (83.1) | 37 (16.9) | 0.71 (0.42-1.20, p=0.199) |
| GGH rs3758149 | GG | 198 (80.5) | 48 (19.5) | - |
|  | AA+GA | 103 (81.7) | 23 (18.3) | 0.92 (0.53-1.60, p=0.770) |
| MTR rs3768142 | GG | 50 (84.7) | 9 (15.3) | - |
|  | TT+GT | 251 (80.2) | 62 (19.8) | 1.37 (0.64-2.94, p=0.416) |
| SLCO1B1 rs4149056 | TT | 241 (79.8) | 61 (20.2) | - |
|  | CC+TC | 60 (85.7) | 10 (14.3) | 0.66 (0.32-1.36, p=0.259) |
| DHFR rs442767 | GG | 44 (84.6) | 8 (15.4) | - |
|  | TT+GT | 255 (80.2) | 63 (19.8) | 1.36 (0.61-3.03, p=0.454) |
| DROSHA rs639174 | CC | 27 (81.8) | 6 (18.2) | - |
|  | TT+CT | 273 (80.8) | 65 (19.2) | 1.07 (0.42-2.70, p=0.884) |
| ABCC2 rs717620 | CC | 193 (82.1) | 42 (17.9) | - |
|  | TT+TC | 108 (78.8) | 29 (21.2) | 1.23 (0.73-2.09, p=0.436) |
| ABCC4 rs7317112 | AA | 139 (80.8) | 33 (19.2) | - |
|  | GG+GA | 161 (80.9) | 38 (19.1) | 0.99 (0.59-1.67, p=0.982) |

**Table S4** Validation of model predictive performance using clinical samples

| **No.** | **Sex^a^** | **MTX dose** | **C_24h_MTX** | **ABCB1 rs1128503** | **SLCO1B1 haplotype** | **Model score^b^** | **Risk^c^ (%)** | **Actual result^d^** | **Accordance^e^** |
| --- | --- | --- | --- | --- | --- | --- | --- | --- | --- |
| 1 | 2 | 2.70 | 1.56 | AA | *1b/*1b | 60 | ＜10 | 0 | H |
| 2 | 2 | 5.00 | 13.86 | AA | *1b/*1b | 68.5 | ＜10 | 0 | H |
| 3 | 2 | 12.00 | 0.12 | AA | *1b/*1b | 100 | 24 | 0 | H |
| 4 | 2 | 4.00 | 10.83 | GG+GA | *1b/*1b | 83.9 | 16 | 0 | H |
| 5 | 2 | 2.86 | 5.72 | GG+GA | *1b/*15 | 62.5 | ＜10 | 0 | H |
| 6 | 2 | 1.40 | 0.51 | AA | *1b/*1b | 48.5 | ＜10 | 0 | H |
| 7 | 1 | 16.00 | 0.09 | AA | *1b/*15 | 78.01 | 14 | 0 | H |
| 8 | 1 | 17.00 | 0.32 | GG+GA | *1b/*1b | 120.03 | 40 | 0 | M |
| 9 | 2 | 1.40 | 0.13 | GG+GA | *1b/*15 | 55.01 | ＜10 | 0 | H |
| 10 | 1 | 2.77 | 2.24 | GG+GA | *1b/*1b | 50.25 | ＜10 | 1 | L |
| 11 | 2 | 5.80 | 12.8175 | AA | *1b/*1b | 70.3 | 11 | 0 | H |
| 12 | 2 | 13.00 | 0.19 | AA | *1b/*1b | 107.02 | 30 | 0 | M |
| 13 | 1 | 12.00 | 0.34 | GG+GA | *1b/*1b | 96.5 | 22 | 0 | H |
| 14 | 1 | 1.80 | 21.3676 | AA | *1b/*1b | 28 | ＜10 | 0 | H |
| 15 | 1 | 1.80 | 4.67 | GG+GA | *1b/*1b | 45.4 | ＜10 | 0 | H |
| 16 | 1 | 1.80 | 0.47 | GG+GA | *1b/*15 | 30.04 | ＜10 | 0 | H |
| 17 | 2 | 1.80 | 1.03 | AA | *1b/*1b | 49.1 | ＜10 | 0 | H |
| 18 | 1 | 1.80 | 1.75 | GG+GA | *1b/*1b | 45.2 | ＜10 | 1 | L |
| 19 | 1 | 1.80 | 0.71 | AA | *1b/*1b | 23.07 | ＜10 | 0 | H |
| 20 | 2 | 1.80 | 10.0838 | AA | *1b/*15 | 34.9 | ＜10 | 0 | H |
| 21 | 1 | 1.80 | 1.09 | GG+GA | *1b/*1b | 45.1 | ＜10 | 0 | H |
| 22 | 1 | 1.80 | 0.27 | GG+GA | *1b/*1b | 45.02 | ＜10 | 0 | H |
| 23 | 1 | 1.80 | 1.19 | GG+GA | *1a/*1a | 85.1 | 18 | 0 | H |
| 24 | 2 | 1.80 | 1 | GG+GA | *1b/*1b | 71.1 | 12 | 0 | H |
| 25 | 1 | 15.00 | 50.72 | GG+GA | *1b/*15 | 105 | 28 | 0 | H |
| 26 | 1 | 1.80 | 0.65 | AA | *1b/*1b | 23.06 | ＜10 | 0 | H |
| 27 | 1 | 6.00 | 13.99 | GG+GA | *1b/*1b | 67.5 | ＜10 | 0 | H |
| 28 | 1 | 15.00 | 0.21 | GG+GA | *1b/*1b | 113.02 | 31 | 1 | M |
| 29 | 2 | 13.00 | 37.61 | AA | *1b/*1b | 112 | 30 | 0 | M |
| 30 | 2 | 15.00 | 0.36 | GG+GA | *1b/*1b | 138.03 | 52 | 0 | L |
| 31 | 2 | 1.80 | 0.24 | GG+GA | *1b/*1b | 71.02 | 11 | 0 | H |
| 32 | 1 | 18.00 | 7.3274 | AA | *1b/*1b | 104.7 | 28 | 0 | H |
| 33 | 2 | 5.00 | 0.4 | GG+GA | *1b/*15 | 63.04 | ＜10 | 0 | H |
| 34 | 1 | 1.80 | 1.18 | AA | *1b/*1b | 23.1 | ＜10 | 0 | H |
| 35 | 1 | 1.80 | 5.42 | GG+GA | *1b/*15 | 30.5 | ＜10 | 0 | H |
| 36 | 1 | 16.00 | 0.11 | AA | *1b/*1b | 94.01 | 22 | 0 | H |
| 37 | 2 | 4.00 | 0.27 | GG+GA | *1b/*1b | 84.02 | 18 | 1 | L |
| 38 | 1 | 1.80 | 2.05 | AA | *1b/*1b | 23.02 | ＜10 | 0 | H |
| 39 | 1 | 1.80 | 0.07 | GG+GA | *1b/*1b | 45 | ＜10 | 0 | H |
| 40 | 1 | 1.80 | 0.52 | AA | *1b/*1b | 23.05 | ＜10 | 0 | H |
| 41 | 1 | 1.80 | 0.96 | AA | *1b/*1b | 23.1 | ＜10 | 0 | H |
| 42 | 2 | 1.80 | 1.01 | AA | *1b/*1b | 49.1 | ＜10 | 0 | H |
| 43 | 1 | 18.00 | 56.2 | GG+GA | *1b/*15 | 121 | 39 | 0 | M |
| 44 | 2 | 1.80 | 9.3066 | AA | *1b/*1b | 50 | ＜10 | 0 | H |
| 45 | 2 | 10.00 | 34.69 | GG+GA | *1b/*1b | 121 | 39 | 0 | M |
| 46 | 2 | 1.80 | 1.1 | GG+GA | *1b/*15 | 56.2 | ＜10 | 0 | H |
| 47 | 1 | 6.00 | 0.56 | GG+GA | *1b/*1b | 66.05 | 11 | 0 | H |
| 48 | 1 | 2.00 | 0.47 | GG+GA | *1b/*1b | 48.05 | ＜10 | 1 | L |
| 49 | 2 | 1.80 | 0.34 | GG+GA | *1b/*1b | 71.03 | 12 | 0 | H |
| 50 | 2 | 16.00 | 275 | AA | *1b/*1b | 169 | 78 | 1 | H |
| 51 | 2 | 1.80 | 24.5 | AA | *1b/*15 | 39 | ＜10 | 0 | H |
| 52 | 1 | 1.80 | 0.87 | AA | *1b/*1b | 23.08 | ＜10 | 0 | H |
| 53 | 2 | 1.50 | 16.6193 | AA | *1a/*1a | 91.5 | 19 | 0 | H |
| 54 | 1 | 1.80 | 0.58 | GG+GA | *1b/*1b | 45.05 | ＜10 | 0 | H |
| 55 | 1 | 7.90 | 2.36 | AA | *1b/*1b | 53.02 | ＜10 | 0 | H |
| 56 | 1 | 16.00 | 19.56 | AA | *1b/*1b | 97.5 | 23 | 0 | H |
| 57 | 1 | 1.80 | 2.7 | GG+GA | *1b/*1b | 45.2 | ＜10 | 0 | H |
| 58 | 1 | 1.80 | 0.27 | GG+GA | *1b/*1b | 45.2 | ＜10 | 0 | H |
| 59 | 1 | 1.80 | 7.45 | AA | *1a/*1a | 63.7 | ＜10 | 0 | H |
| 60 | 1 | 18.00 | 16.12 | GG+GA | *1b/*15 | 114.5 | 35 | 0 | M |
| 61 | 1 | 8.00 | 0.49 | GG+GA | *1b/*1b | 46.05 | ＜10 | 1 | L |
| 62 | 1 | 3.00 | 1.32 | AA | *1b/*15 | 15.1 | ＜10 | 0 | H |
| 63 | 1 | 15.00 | 0.31 | AA | *1b/*1b | 91.03 | 19 | 1 | L |
| 64 | 1 | 1.80 | 0.28 | GG+GA | *1b/*1b | 45.02 | ＜10 | 0 | H |
| 65 | 1 | 1.80 | 8.08 | GG+GA | *1a/*1a | 85.8 | 27 | 0 | H |
| 66 | 2 | 1.80 | 0.36 | GG+GA | *1b/*1b | 71.03 | 11 | 0 | H |
| 67 | 2 | 1.80 | 1.02 | AA | *1b/*1b | 49.1 | ＜10 | 0 | H |
| 68 | 1 | 1.80 | 0.27 | GG+GA | *1b/*1b | 45.02 | ＜10 | 0 | H |
| 69 | 1 | 18.00 | 0.2 | GG+GA | *1b/*1b | 127.02 | 48 | 1 | M |
| 70 | 2 | 1.80 | 10.5 | GG+GA | *1b/*15 | 57 | ＜10 | 0 | H |
| 71 | 2 | 1.80 | 0.45 | GG+GA | *1a/*1a | 111.05 | 31 | 0 | M |
| 72 | 1 | 1.80 | 1.06 | GG+GA | *1b/*15 | 30.1 | ＜10 | 0 | H |
| 73 | 1 | 7.45 | 0.54 | GG+GA | *1b/*1b | 74.05 | 13 | 0 | H |
| 74 | 1 | 1.80 | 5.79 | AA | *1b/*15 | 8.5 | ＜10 | 0 | H |
| 75 | 1 | 18.00 | 0.27 | AA | *1b/*1b | 104.02 | 29 | 0 | H |
| 76 | 2 | 1.72 | 0.07 | AA | *1b/*1b | 49 | ＜10 | 0 | H |
| 77 | 2 | 1.80 | 0.61 | GG+GA | *1b/*1b | 71.06 | 12 | 0 | H |
| 78 | 2 | 1.80 | 0.43 | GG+GA | *1a/*1a | 111.04 | 32 | 0 | M |
| 79 | 2 | 1.80 | 0.85 | GG+GA | *1b/*1b | 71.08 | 12 | 0 | H |
| 80 | 1 | 1.90 | 0.22 | AA | *1b/*1b | 23.02 | ＜10 | 0 | H |
| 81 | 2 | 5.00 | 0.26 | GG+GA | *1b/*1b | 88.02 | 19 | 0 | H |
| 82 | 2 | 1.57 | 0.44 | AA | *1b/*1b | 48.04 | ＜10 | 0 | H |
| 83 | 2 | 12.00 | 2.83 | GG+GA | *1b/*1b | 123.2 | 41 | 0 | M |
| 84 | 1 | 12.00 | 0.15 | GG+GA | *1b/*15 | 82.01 | 15 | 0 | H |
| 85 | 1 | 5.00 | 1.17 | GG+GA | *1b/*1b | 62.1 | ＜10 | 0 | H |
| 86 | 1 | 0.11 | 0.29 | GG+GA | *1b/*1b | 38.02 | ＜10 | 0 | H |
| 87 | 1 | 17.00 | 10.19 | GG+GA | *1b/*1b | 125 | 45 | 1 | M |
| 88 | 1 | 15.00 | 0.2 | GG+GA | *1b/*1b | 112.02 | 31 | 0 | M |
| 89 | 2 | 4.60 | 6.78 | AA | *1b/*1b | 64.6 | ＜10 | 0 | H |
| 90 | 2 | 1.80 | 0.49 | GG+GA | *1b/*15 | 56.05 | ＜10 | 0 | H |
| 91 | 2 | 13.00 | 32.78 | GG+GA | *1b/*1b | 136 | 51 | 0 | L |
| 92 | 2 | 4.00 | 7.4252 | AA | *1b/*1b | 61.7 | ＜10 | 0 | H |
| 93 | 1 | 2.10 | 0.13 | GG+GA | *1b/*1b | 48.1 | ＜10 | 0 | H |
| 94 | 1 | 18.00 | 0.16 | GG+GA | *1b/*1b | 126.01 | 44 | 0 | M |
| 95 | 1 | 1.80 | 2.14 | GG+GA | *1b/*1b | 45.2 | ＜10 | 0 | H |
| 96 | 1 | 15.00 | 14.88 | AA | *1b/*1b | 91.5 | 19 | 1 | L |
| 97 | 1 | 12.00 | 0.6 | GG+GA | *1b/*1b | 98.06 | 25 | 1 | L |
| 98 | 1 | 1.80 | 0.14 | GG+GA | *1b/*1b | 45.01 | ＜10 | 0 | H |
| 99 | 1 | 15.00 | 10.94 | GG+GA | *1b/*15 | 97.1 | 24 | 0 | H |
| 100 | 1 | 1.80 | 0.38 | AA | *1b/*1b | 23.03 | ＜10 | 0 | H |
| 101 | 1 | 1.80 | 0.47 | GG+GA | *1b/*15 | 30.04 | ＜10 | 0 | H |
| 102 | 2 | 13.00 | 0.11 | GG+GA | *1b/*1b | 128.01 | 48 | 0 | M |
| 103 | 1 | 1.80 | 0.33 | GG+GA | *1b/*1b | 45.03 | ＜10 | 0 | H |
| 104 | 1 | 1.80 | 0.22 | AA | *1b/*15 | 8.02 | ＜10 | 0 | H |
| 105 | 1 | 1.80 | 11.76 | GG+GA | *1b/*1b | 46 | ＜10 | 0 | H |
| 106 | 1 | 1.80 | 0.09 | AA | *1b/*1b | 23 | ＜10 | 0 | H |
| 107 | 1 | 1.80 | 3.19 | GG+GA | *1b/*1b | 45.3 | ＜10 | 0 | H |
| 108 | 1 | 1.80 | 0.44 | AA | *1b/*1b | 23.04 | ＜10 | 0 | H |
| 109 | 2 | 4.43 | 0.56 | AA | *1b/*1b | 64.05 | 11 | 1 | L |
| 110 | 1 | 14.00 | 0.24 | AA | *1b/*1b | 85.02 | 18 | 1 | L |
| 111 | 2 | 1.50 | 3.0507 | AA | *1a/*1a | 88.3 | 19 | 0 | H |
| 112 | 1 | 18.00 | 70.16 | GG+GA | *1b/*1b | 144 | 60 | 0 | L |
| 113 | 2 | 1.56 | 2.22 | GG+GA | *1b/*1b | 70.7 | 11 | 0 | H |
| 114 | 1 | 1.56 | 1.11 | GG+GA | *1b/*1b | 44.6 | ＜10 | 0 | H |
| 115 | 2 | 1.58 | 0.07 | GG+GA | *1b/*1b | 70.5 | 11 | 0 | H |
| 116 | 1 | 3.35 | 0.36 | GG+GA | *1b/*1b | 53.03 | ＜10 | 0 | H |
| 117 | 2 | 10.00 | 0.06 | GG+GA | *1b/*15 | 98 | 25 | 1 | L |
| 118 | 1 | 16.00 | 8.7477 | GG+GA | *1b/*1b | 116.8 | 31 | 0 | M |
| 119 | 2 | 13.00 | 21.33 | AA | *1b/*15 | 97 | 24 | 1 | L |
| 120 | 2 | 1.50 | 10.0817 | AA | *1a/*1a | 89.5 | 20 | 0 | H |
| 121 | 2 | 1.19 | 2.17 | AA | *1b/*1b | 48.2 | ＜10 | 1 | L |
| 122 | 1 | 15.00 | 0.11 | AA | *1b/*15 | 75.01 | 13 | 0 | H |
| 123 | 1 | 14.00 | 3.12 | GG+GA | *1b/*1b | 106.3 | 28 | 0 | H |
| 124 | 2 | 17.00 | 0.1 | GG+GA | *1b/*1b | 149.01 | 64 | 1 | H |
| 125 | 1 | 4.00 | 0.5 | AA | *1b/*15 | 20.05 | ＜10 | 0 | H |
| 126 | 2 | 13.00 | 26.1 | GG+GA | *1b/*1b | 134.5 | 50 | 1 | H |
| 127 | 1 | 2.60 | 1.43 | GG+GA | *1b/*1b | 50.1 | ＜10 | 0 | H |
| 128 | 1 | 2.25 | 0.17 | GG+GA | *1a/*1a | 90.01 | 19 | 1 | L |
| 129 | 1 | 15.00 | 0.09 | AA | *1b/*1b | 90 | 19 | 0 | H |
| 130 | 1 | 2.00 | 0.84 | GG+GA | *1b/*15 | 32.08 | ＜10 | 0 | H |
| 131 | 1 | 2.10 | 35 | AA | *1b/*1b | 34 | ＜10 | 0 | H |
| 132 | 1 | 14.00 | 12.5574 | GG+GA | *1b/*1b | 108.1 | 31 | 0 | M |
| 133 | 1 | 3.94 | 0.68 | GG+GA | *1b/*1b | 55.06 | ＜10 | 0 | H |
| 134 | 2 | 5.00 | 0.22 | AA | *1b/*15 | 51.02 | ＜10 | 0 | H |
| 135 | 1 | 4.72 | 0.2 | GG+GA | *1b/*1b | 60.02 | ＜10 | 0 | H |
| 136 | 1 | 10.00 | 63.23 | AA | *1b/*15 | 64 | ＜10 | 0 | H |
| 137 | 1 | 15.00 | 15.89 | AA | *1b/*1b | 91.5 | 19 | 0 | H |
| 138 | 1 | 2.00 | 1.35 | GG+GA | *1b/*1b | 47.1 | ＜10 | 0 | H |
| 139 | 2 | 16.00 | 24.18 | AA | *1b/*1b | 126 | 45 | 1 | M |
| 140 | 1 | 1.93 | 0.57 | AA | *1b/*1b | 14.05 | ＜10 | 0 | H |
| 141 | 1 | 4.80 | 11.87 | GG+GA | *1b/*1b | 61.1 | ＜10 | 0 | H |
| 142 | 1 | 3.60 | 0.05 | GG+GA | *1b/*15 | 40 | ＜10 | 0 | H |
| 143 | 2 | 4.10 | 17.1939 | GG+GA | *1b/*15 | 71 | 12 | 0 | H |
| 144 | 1 | 18.00 | 0.13 | GG+GA | *1b/*1b | 127.01 | 46 | 0 | M |
| 145 | 2 | 5.00 | 1.05 | GG+GA | *1b/*1b | 88.1 | 19 | 0 | H |
| 146 | 2 | 12.00 | 27.15 | AA | *1b/*1b | 105.5 | 29 | 0 | H |
| 147 | 1 | 15.00 | 116.7 | GG+GA | *1b/*1b | 135 | 51 | 0 | L |
| 148 | 2 | 3.90 | 2.58 | AA | *1b/*1b | 61.2 | ＜10 | 0 | H |
| 149 | 2 | 3.70 | 3.3942 | AA | *1a/*1a | 100.3 | 24 | 1 | L |
| 150 | 1 | 1.75 | 0.75 | AA | *1b/*1b | 23.07 | ＜10 | 0 | H |
| 151 | 2 | 6.50 | 3.3 | GG+GA | *1b/*15 | 83 | 17 | 1 | L |
| 152 | 1 | 15.00 | 0.14 | GG+GA | *1b/*15 | 7.01 | ＜10 | 0 | H |
| 153 | 1 | 5.00 | 0.18 | GG+GA | *1b/*1b | 62.01 | ＜10 | 0 | H |
| 154 | 1 | 8.00 | 8.15 | AA | *1b/*15 | 40.8 | ＜10 | 0 | H |
| 155 | 1 | 4.35 | 0.23 | GG+GA | *1b/*1b | 46.02 | ＜10 | 0 | H |
| 156 | 1 | 12.00 | 0.1 | GG+GA | *1b/*15 | 81.01 | 15 | 0 | H |
| 157 | 2 | 13.00 | 0.72 | GG+GA | *1b/*1b | 127.07 | 46 | 1 | M |
| 158 | 2 | 15.00 | 198.41 | GG+GA | *1b/*1b | 173 | 78 | 1 | H |
| 159 | 1 | 1.85 | 0.6 | GG+GA | *1b/*15 | 30.06 | ＜10 | 0 | H |
| 160 | 1 | 1.60 | 0.31 | AA | *1b/*15 | 7.53 | ＜10 | 0 | H |
| 161 | 2 | 18.00 | 0.93 | GG+GA | *1b/*1b | 152.09 | 64 | 0 | L |
| 162 | 2 | 3.72 | 0.49 | GG+GA | *1b/*15 | 66.05 | 11 | 0 | H |
| 163 | 1 | 2.88 | 0.45 | AA | *1b/*1b | 50.05 | ＜10 | 0 | H |
| 164 | 1 | 1.75 | 0.5 | AA | *1b/*1b | 23.05 | ＜10 | 0 | H |
| 165 | 2 | 4.07 | 11.91 | GG+GA | *1b/*15 | 69.1 | 12 | 0 | H |
| 166 | 1 | 3.33 | 0.37 | GG+GA | *1b/*15 | 38.03 | ＜10 | 0 | H |
| 167 | 1 | 1.75 | 0.82 | GG+GA | *1a/*1a | 85.08 | 17 | 0 | H |
| 168 | 1 | 5.65 | 0.65 | AA | *1b/*1b | 42.06 | ＜10 | 0 | H |
| 169 | 1 | 2.90 | 0.5 | GG+GA | *1b/*1b | 52.05 | ＜10 | 0 | H |
| 170 | 1 | 3.60 | 0.24 | GG+GA | *1b/*1b | 54.02 | ＜10 | 0 | H |
| 171 | 1 | 4.26 | 0.74 | AA | *1b/*1b | 37.07 | ＜10 | 0 | H |
| 172 | 1 | 12.00 | 0.51 | GG+GA | *1b/*1b | 96.05 | 22 | 0 | H |
| 173 | 2 | 3.00 | 28.75 | AA | *1a/*1a | 103 | 24 | 0 | H |
| 174 | 2 | 12.00 | 8.066 | AA | *1a/*1a | 141.8 | 55 | 1 | H |
| 175 | 2 | 5.50 | 0.21 | GG+GA | *1b/*1b | 90.02 | 19 | 0 | H |
| 176 | 2 | 2.95 | 0.34 | GG+GA | *1b/*1b | 78.03 | 14 | 0 | H |
| 177 | 2 | 17.00 | 0.27 | GG+GA | *1b/*1b | 149.02 | 63 | 1 | H |
| 178 | 1 | 5.00 | 0.03 | GG+GA | *1b/*15 | 47 | ＜10 | 0 | H |
| 179 | 1 | 5.30 | 6.09 | GG+GA | *1b/*1b | 63.6 | ＜10 | 0 | H |
| 180 | 2 | 1.30 | 23.2528 | GG+GA | *1b/*15 | 56.5 | ＜10 | 0 | H |
| 181 | 2 | 4.00 | 21.8138 | AA | *1b/*1b | 64 | ＜10 | 0 | H |
| 182 | 1 | 14.00 | 13.88 | GG+GA | *1b/*1b | 108.3 | 30 | 1 | M |
| 183 | 1 | 6.00 | 11.2754 | GG+GA | *1b/*15 | 52.1 | ＜10 | 0 | H |
| 184 | 2 | 1.50 | 7.65 | AA | *1b/*1b | 49.2 | ＜10 | 0 | H |
| 185 | 1 | 4.53 | 3.05 | AA | *1a/*1a | 78.3 | 14 | 0 | H |
| 186 | 1 | 5.70 | 13.27 | AA | *1b/*1b | 43.3 | ＜10 | 0 | H |
| 187 | 1 | 8.00 | 2.57 | AA | *1b/*1b | 54.2 | ＜10 | 0 | H |
| 188 | 1 | 15.00 | 50.91 | GG+GA | *1b/*15 | 106 | 30 | 1 | M |
| 189 | 1 | 12.00 | 4.2 | GG+GA | *1b/*1b | 86.4 | 18 | 0 | H |
| 190 | 2 | 11.00 | 1.57 | GG+GA | *1b/*1b | 119.1 | 40 | 1 | M |
| 191 | 2 | 4.70 | 13.62 | GG+GA | *1b/*1b | 88.3 | 18 | 0 | H |
| 192 | 1 | 15.00 | 9.7178 | AA | *1b/*1b | 90.9 | 20 | 0 | H |
| 193 | 2 | 3.89 | 0.83 | GG+GA | *1b/*1b | 82.8 | 16 | 0 | H |
| 194 | 2 | 0.51 | 0.36 | AA | *1b/*1b | 43.53 | ＜10 | 0 | H |
| 195 | 1 | 3.20 | 1.04 | GG+GA | *1b/*1b | 53.1 | ＜10 | 0 | H |
| 196 | 1 | 2.11 | 3 | GG+GA | *1b/*15 | 33.3 | ＜10 | 0 | H |
| 197 | 1 | 1.80 | 0.53 | GG+GA | *1b/*1b | 45.05 | ＜10 | 0 | H |
| 198 | 1 | 1.90 | 24.0454 | AA | *1b/*15 | 13 | ＜10 | 0 | H |
| 199 | 2 | 15.00 | 37.75 | GG+GA | *1b/*1b | 146 | 61 | 1 | H |
| 200 | 2 | 1.50 | 0.37 | AA | *1b/*1b | 48.03 | ＜10 | 0 | H |
| 201 | 1 | 2.00 | 11.719 | GG+GA | *1b/*15 | 33.1 | ＜10 | 0 | H |
| 202 | 2 | 13.00 | 0.26 | GG+GA | *1b/*1b | 128.02 | 46 | 0 | M |
| 203 | 1 | 3.45 | 1.23 | GG+GA | *1b/*1b | 54.1 | ＜10 | 0 | H |
| 204 | 1 | 1.50 | 0.2 | AA | *1b/*1b | 22.02 | ＜10 | 0 | H |
| 205 | 1 | 6.60 | 4.28 | GG+GA | *1b/*1b | 59.4 | ＜10 | 0 | H |
| 206 | 2 | 5.00 | 0.25 | GG+GA | *1b/*15 | 73.02 | 12 | 0 | H |
| 207 | 2 | 4.50 | 2.69 | GG+GA | *1b/*1b | 87.2 | 18 | 0 | H |
| 208 | 2 | 13.00 | 0.39 | AA | *1b/*1b | 106.03 | 28 | 0 | H |
| 209 | 1 | 19.00 | 27.5 | GG+GA | *1b/*1b | 136.5 | 50 | 0 | L |
| 210 | 2 | 14.00 | 40.75 | GG+GA | *1b/*1b | 142 | 56 | 1 | H |
| 211 | 1 | 2.89 | 0.35 | AA | *1b/*1b | 30.03 | ＜10 | 0 | H |
| 212 | 1 | 4.00 | 7.6047 | AA | *1b/*1b | 35.7 | ＜10 | 0 | H |
| 213 | 1 | 1.80 | 9.0542 | GG+GA | *1b/*1b | 45.9 | ＜10 | 0 | H |
| 214 | 1 | 2.63 | 0.23 | GG+GA | *1b/*1b | 52.02 | ＜10 | 0 | H |
| 215 | 2 | 5.00 | 0.16 | AA | *1b/*1b | 66.01 | 11 | 0 | H |
| 216 | 1 | 10.00 | 8.76 | AA | *1b/*1b | 64.8 | 11 | 0 | H |
| 217 | 2 | 4.74 | 7.73 | AA | *1b/*1b | 72 | 13 | 0 | H |
| 218 | 1 | 16.00 | 10.13 | AA | *1b/*1b | 95 | 21 | 0 | H |
| 219 | 2 | 3.21 | 1.07 | AA | *1b/*1b | 57.1 | ＜10 | 0 | H |
| 220 | 2 | 13.00 | 57.12 | GG+GA | *1b/*1b | 141 | 56 | 1 | H |
| 221 | 1 | 3.00 | 0.12 | GG+GA | *1b/*1b | 52.01 | ＜10 | 0 | H |
| 222 | 1 | 4.42 | 4.43 | GG+GA | *1b/*1b | 61.4 | ＜10 | 0 | H |
| 223 | 1 | 1.50 | 0.3 | GG+GA | *1b/*15 | 29.03 | ＜10 | 0 | H |
| 224 | 1 | 2.00 | 0.36 | GG+GA | *1b/*1b | 47.03 | ＜10 | 0 | H |
| 225 | 1 | 2.55 | 0.68 | GG+GA | *1b/*1b | 51.6 | ＜10 | 1 | L |
| 226 | 1 | 9.00 | 0.09 | AA | *1b/*1b | 60 | ＜10 | 0 | H |
| 227 | 2 | 13.00 | 10.16 | GG+GA | *1b/*1b | 109 | 31 | 0 | M |
| 228 | 1 | 18.00 | 0.21 | GG+GA | *1b/*15 | 112.02 | 32 | 1 | M |
| 229 | 2 | 4.50 | 8.16 | GG+GA | *1b/*1b | 86.8 | 17 | 0 | H |
| 230 | 2 | 15.00 | 0.15 | AA | *1b/*1b | 117.01 | 35 | 1 | M |
| 231 | 2 | 12.00 | 0.56 | GG+GA | *1b/*1b | 122.05 | 41 | 0 | M |
| 232 | 1 | 11.00 | 9.5467 | GG+GA | *1b/*1b | 93.9 | 19 | 1 | L |
| 233 | 2 | 1.35 | 19.7582 | AA | *1b/*1b | 51.5 | ＜10 | 0 | H |
| 234 | 2 | 13.00 | 40.0194 | AA | *1b/*1b | 114 | 35 | 0 | M |
| 235 | 2 | 5.00 | 7.34 | GG+GA | *1b/*1b | 89.7 | 19 | 1 | L |
| 236 | 2 | 1.50 | 1.73 | GG+GA | *1b/*1b | 70.6 | 11 | 0 | H |
| 237 | 2 | 8.00 | 0.16 | AA | *1b/*1b | 80.01 | 14 | 0 | H |

**a**, 1 means male, 2 means female; **b**, Model score means the overall point calculated from logistic regression nomogram; **c**, Risk of developing moderate/severe DILI according to the overall predictive point; **d**, 1 means patient with moderate or severe DILI; 0 means patients with no moderate or severe DILI; **e**, H means the accordance of predictive risk probability and actual result was high (over 50% accord with 1, lower than 30% accord with 0); M means the accordance was in the middle (30%~50% accord with 1, 0); L means the accordance was low (lower than 30% accord with 1, over 50% accord with 0).


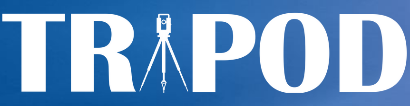


| **Section/Topic** | **Item** | **Checklist Item** | **Page** | **Text Excerpt** |
| --- | --- | --- | --- | --- |
| **Title and abstract** | | | |  |
| Title | 1 | Identify the study as developing and/or validating a multivariable prediction model, the target population, and the outcome to be predicted. | 1 | “Integration of genomics, clinical characteristics and baseline biological profiles to predict the risk of liver injury induced by high-dose methotrexate” |
| Abstract | 2 | Provide a summary of objectives, study design, setting, participants, sample size, predictors, outcome, statistical analysis, results, and conclusions. | 2 | Objectives/study design/setting/participants: High-dose methotrexate (HD-MTX) is commonly employed in […] spectrometry analysis.  Sample size: N=374  Predictors: Large number pf predictors which were not included in abstract can be seen in page 5/6  Outcome: drug induced liver injury (DILI)  Statistical analysis: “establish a predictive model in the form of a nomogram that integrates genetic biomarkers and clinimetric markers”  Result: “G allele mutation […] exhibited high accuracy. “  Conclusion: “Female patients […] proactive prevention strategies.” |
| **Introduction** | | | |  |
| Background and objectives | 3a | Explain the medical context (including whether diagnostic or prognostic) and rationale for developing or validating the multivariable prediction model, including references to existing models. | 4 | “Consequently, individual differences in efficacy […] rescue opportunity and inducing toxicity” |
|  | 3b | Specify the objectives, including whether the study describes the development or validation of the model or both. | 4 | “Therefore, the objective of this study is to establish a diagnostic predictive model […] stages of hospitalization.” |
| **Methods** | | | |  |
| Source of data | 4a | Describe the study design or source of data (e.g., randomized trial, cohort, or registry data), separately for the development and validation data sets, if applicable. | 5 | “Retrospective data for model development were extracted from the medical record system before and after treatment, including demographic data […] drugs interactions).” |
|  | 4b | Specify the key study dates, including start of accrual; end of accrual; and, if applicable, end of follow-up. | 5 | “A total of 374 patients […] from December 2019, to July 2022.” |
| Participants | 5a | Specify key elements of the study setting (e.g., primary care, secondary care, general population) including number and location of centres. | 5 | We included “hospitalized patients firstly and newly diagnosed with ALL, NHL, OS” in one center. |
|  | 5b | Describe eligibility criteria for participants. | 5 | “The inclusion criteria were (1) hospitalized patients firstly and newly diagnosed with ALL, NHL, OS or Langerhans’cell histiocytosis; (2) patients who […] after initial administration. |
|  | 5c | Give details of treatments received, if relevant. | 6 | “In the treatment of hematologic malignancies […] lasting 4–6 hours” |
| Outcome | 6a | Clearly define the outcome that is predicted by the prediction model, including how and when assessed. | 5 | “Liver functional indicators were used to determine DILI […] if baseline was abnormal.” |
|  | 6b | Report any actions to blind assessment of the outcome to be predicted. | NA | NA |
| Predictors | 7a | Clearly define all predictors used in developing or validating the multivariable prediction model, including how and when they were measured. | 5/7 | “Retrospective data for model development were extracted from the medical record system before and after treatment, including demographic data […] drugs interactions).” “We selected 25 SNPs as our target gene sequence (Table 2).” |
|  | 7b | Report any actions to blind assessment of predictors for the outcome and other predictors. | NA | NA |
| Sample size | 8 | Explain how the study size was arrived at. | NA | NA |
| Missing data | 9 | Describe how missing data were handled (e.g., complete-case analysis, single imputation, multiple imputation) with details of any imputation method. | 7 | we meticulously documented any instances of missing data […] of missing values |
| Statistical analysis methods | 10a | Describe how predictors were handled in the analyses. | NA | NA |
|  | 10b | Specify type of model, all model-building procedures (including any predictor selection), and method for internal validation. | 7/8 | “The stepwise regression analysis (backward method) […] to visualize predictions for complex models.” |
|  | 10d | Specify all measures used to assess model performance and, if relevant, to compare multiple models. | 8 | “Finally, the efficiency and clinical significance of the predictive model was evaluated […] was used to generate figures.” |
| Risk groups | 11 | Provide details on how risk groups were created, if done. | NA | NA |
| **Results** | | | |  |
| Participants | 13a | Describe the flow of participants through the study, including the number of participants with and without the outcome and, if applicable, a summary of the follow-up time. A diagram may be helpful. | 8/9 | Fig. 1. Flowchart of patient inclusion and statistical analysis process |
|  | 13b | Describe the characteristics of the participants (basic demographics, clinical features, available predictors), including the number of participants with missing data for predictors and outcome. | 8/9 | “Baseline demographic and pharmaceutical parameters […] in the none/mild DILI cohort.” |
| Model development | 14a | Specify the number of participants and outcome events in each analysis. | 8 | “Ultimately, 374 eligible patients were enrolled […] triple Intrathecal injection |
|  | 14b | If done, report the unadjusted association between each candidate predictor and outcome. | 8/9/10/11 | The specific association between predictors and outcome could be seen in part 3.1 and 3.3 |
| Model specification | 15a | Present the full prediction model to allow predictions for individuals (i.e., all regression coefficients, and model intercept or baseline survival at a given time point). | 11 | “The following factors were selected into binary logistic regression model […] predict the probability of outcome based on total scores:” The specific parameters of model were showed in table 4 |
|  | 15b | Explain how to the use the prediction model. | NA | NA |
| Model performance | 16 | Report performance measures (with CIs) for the prediction model. | 11 | Reported |
| **Discussion** | | | |  |
| Limitations | 18 | Discuss any limitations of the study (such as nonrepresentative sample, few events per predictor, missing data). | 16 | “While the study presents significant findings, […] impact on patient care.” |
| Interpretation | 19b | Give an overall interpretation of the results, considering objectives, limitations, and results from similar studies, and other relevant evidence. | 12/13 | “This retrospective longitudinal study […] developing predictive models for clinical toxicity.” |
| Implications | 20 | Discuss the potential clinical use of the model and implications for future research. | 15/16 | “Overall, nomogram provides a visual […] may prompt preemptive interventions.” |
| **Other information** | | | |  |
| Supplementary information | 21 | Provide information about the availability of supplementary resources, such as study protocol, Web calculator, and data sets. | Supplementary materials | NA |
| Funding | 22 | Give the source of funding and the role of the funders for the present study. | 17 | This work was supported in […] New Clinical Medical Technology Project of the Second Xiangya Hospital of Central South University ([2021]94). |

We recommend using the TRIPOD Checklist in conjunction with the TRIPOD Explanation and Elaboration document.
